# Laparoscopy in management of appendicitis in high-, middle-, and low-income countries: a multicenter, prospective, cohort study

**DOI:** 10.1007/s00464-018-6064-9

**Published:** 2018-04-05

**Authors:** GlobalSurg Collaborative, Thomas M. Drake, Julian Camilleri-Brennan, Stephen Tabiri, Stuart J. Fergusson, Richard Spence, J. Edward F. Fitzgerald, Aneel Bhangu, Ewen M. Harrison, Adesoji O. Ademuyiwa, Stuart Fergusson, James C. Glasbey, Chetan Khatri, Midhun Mohan, Dmitri Nepogodiev, Kjetil Søreide, Neel Gobin, Ana Vega Freitas, Nigel Hall, Sung-Hee Kim, Ahmed Negida, Zahra Jaffry, Stephen J. Chapman, Alexis P. Arnaud, Gustavo Recinos, Cutting Edge Manipal, Radhian Amandito, Marwan Shawki, Michael Hanrahan, Francesco Pata, Justas Zilinskas, April Camilla Roslani, Cheng Chun Goh, Gareth Irwin, Sebastian Shu, Laura Luque, Hunain Shiwani, Afnan Altamimi, Mohammed Ubaid Alsaggaf, Sarah Rayne, Jenifa Jeyakumar, Yucel Cengiz, Dmitri A. Raptis, Claudio Fermani, Ruben Balmaceda, Maria Marta Modolo, Ewan Macdermid, Roxanne Chenn, Cheryl Ou Yong, Michael Edye, Martin Jarmin, Scott K. D’amours, Dushyant Iyer, Daniel Youssef, Nicholas Phillips, Jason Brown, Robert George, Cherry Koh, Oliver Warren, Isaac Hanley, Marilla Dickfos, Clemens Nawara, Dietmar Öfner, Florian Primavesi, Ashrarur Rahman Mitul, Khalid Mahmud, Margub Hussain, Hafiz Hakim, Tapan Kumar, Antje Oosterkamp, Pamphile A. Assouto, Ismail Lawani, Yacoubou Imorou Souaibou, Aung Kyaw Tun, Chean Leung Chong, Giridhar H. Devadasar, Muhammad Rashid Minhas Qadir, Kyaw Phyo Aung, Lee Shi Yeo, Vanessa Dina Palomino Castillo, Monique Moron Munhoz, Gisele Moreira, Luiz Carlos Barros De Castro Segundo, Salim Anderson Khouri Ferreira, Maíra Cassa Careta, Stella Binna Kim, Alexandre Venancio De Sousa, Alyne Daltri Lazzarini Cury, Gustavo Peixoto Soares Miguel, Ana Vega Carreiro De Freitas, Barbara Pereira Silvestre, Julia Guasti Pinto Vianna, Carolina Oliveira Felipe, Luis Alberto Valente Laufer, Fernanda Altoe, Luana Ayres Da Silva, Marina Luiza Pimenta, Thiago Fernandes Giuriato, Paulo Alves Bezerra Morais, Jessica Souza Luiz, Rafael Araujo, Juliana Menegussi, Marisa Leal, Caio Vinícius Barroso de Lima, Luiza Sarmento Tatagiba, Antônio Leal, Diogo Vinicius dos Santos, Gustavo Pereira Fraga, Romeo Lages Simoes, Simon Stock, Samuel Nigo, Juana Kabba, Tagang Ebogo Ngwa, James Brown, Sebastian King, Augusto Zani, Georges Azzie, Mohammed Firdouse, Sameer Kushwaha, Arnav Agarwal, Karen Bailey, Brian Cameron, Michael Livingston, Alexandre Horobjowsky, Dan L. Deckelbaum, Tarek Razek, Boris Marinkovic, Eugenio Grasset, Nicole D’aguzan, Julio Jimenez, Roberto Macchiavello, Zhongtao Zhang, Wei Guo, Junyeong Oh, Fei Zheng, Irene Montes, Sebastian Sierra, Manuela Mendez, Maria Isabel Villegas, Maria Clara Mendoza Arango, Ivan Mendoza, Fred Alexander Naranjo Aristizã¡bal, Jaime Andres Montoya Botero, Victor Manuel Quintero Riaza, Jakeline Restrepo, Carlos Morales, Herman Cruz, Alejandro Munera, Robert Karlo, Edgar Domini, Jakov Mihanovic, Mihael Radic, Kresimir Zamarin, Nikica Pezelj, Manuel Hache-Marliere, Sylvia Batista Lemaire, Ruben Rivas, Ahmed Khyrallh, Ahamed Hassan, Gamal Shimy, Mohamed A. Baky Fahmy, Ayman Nabawi, Mohamed Elfil, Mohamed Ghoneem, Muhammad El-Saied Ahmad Muhammad Gohar, Mohamed Asal, Mostafa Abdelkader, Mahmoud Gomah, Hayssam Rashwan, Mohamed Karkeet, Ahmed Gomaa, Amr Hasan, Ahmed Elgebaly, Omar Saleh, Ahmad Abdel Fattah, Abdullah Gouda, Abd Elrahman Elshafay, Abdalla Gharib, Ahmed Menshawy, Mohammed Hanafy, Abdullah Al-Mallah, Mahmoud Abdulgawad, Mohamad Baheeg, Mohammed Alhendy, Ibrahim AbdelFattah, Abdalla Kenibar, Omar Osman, Mostafa Gemeah, Ahmed Mohammed, Abdalrahman Adel, Abdelrahman Mohammed, Abdelrahman Sayed, Mohamed Abozaid, Ahmed Hafez El-Badri Kotb, Ali Amin Ahmed Ata, Mohammed Nasr, Abdelrahman Alkammash, Mohammed Saeed, Nader Abd El Hamid, Attia Mohamed Attia, Ahmed Abd El Galeel, Eslam Elbanby, Khalid Salah El-Dien, Usama Hantour, Omar Alahmady, Billal Mansour, Amr Muhammad Elkorashy, Emad Mohamed Saeed Taha, Kholod Tarek Lasheen, Salma Said Elkolaly, Nehal Yosri Elsayed Abdel-Wahab, Mahmoud Ahmed Fathi Abozyed, Ahmed Adel, Ahmed Moustafa Saeed, Gehad Samir El Sayed, Jehad Hassan Youssif, Soliman Magdy Ahmed, Nermeen Soubhy El-Shahat, Abd El-Rahman Hegazy Khedr, Abdelrhman Osama Elsebaaye, Mohamed Elzayat, Mohamed Abdelraheim, Ibrahim Elzayat, Mahmoud Warda, Khaled Naser El Deen, Abdelrhman Essam Elnemr, Omar Salah, Mohamed Abbas, Mona Rashad, Ibrahim Elzayyat, Dalia Hemeda, Gehad Tawfik, Mai Salama, Hazem Khaled, Mohamed Seisa, Kareem Elshaer, Abdelfatah Hussein, Mahmoud Elkhadrawi, Ahmed Mohamed Afifi, Osama Saadeldeen Ebrahim, Mahmoud Mohamed Metwally, Rowida Elmelegy, Diaa Moustafa Elbendary Elsawahly, Hisham Safa, Eman Nofal, Mohamed Elbermawy, Ahmed Abdelmotaleb Ghazy, Hisham Samih, Asmaa Abdelgelil, Sarah Abdelghany, Ahmed El Kholy, Metwally Aboraya, Fatma Elkady, Mahmoud Salma, Sarah Samy, Reem Fakher, Aya Aboarab, Ahmed Samir, Ahmed Sakr, Abdelrahman Haroun, Asmaa Abdel-Rahman Al-Aarag, Ahmed Elkholy, Sally Elshanwany, Esraa Ghanem, Ahmed Tammam, Ali Mohamed Hammad, Yousra El Shoura, Gehad El Ashal, Hosni Khairy, Sarah Antar, Sara Mehrez, Mahmoud Abdelshafy, Maha Gamal Mohamad Hamad, Mona Hosh, Emad Abdallah, Basma Magdy, Thuraya Alzayat, Elsayed Gamaly, Hossam Elfeki, Amany Abouzahra, Shereen Elsheikh, Fatimah I. Elgendy, Fathia Abd El-Salam, Osama Seifelnasr, Mohamed Ammar, Athar Eysa, Aliaa Sadek, Aliaa Gamal Toeema, Aly Nasr, Mohamed Abuseif, Hagar Zidan, Sara Abd Elmageed Barakat, Nadin Elsayed, Yasmin Abd Elrasoul, Ahmed Elkelany, Mohamed Sabry Ammar, Mennat-Allah Mustafa, Yasmin Hegazy, Mohamed Etman, Samar Saad, Mahmoud Alrahawy, Ahmed Raslan, Mahmoud Morsi, Ahmed Rslan, Ahmed Sabry, Hager Elwakil, Heba Shaker, Yasmin Abd-Elrasoul, Hussein El-Kashef, Mohamed Shaalan, Areej Tarek, Ayman Elwan, Ahmed Ragab Nayel, Mostafa Seif, Doaa Emadeldin, Mohamed Ali Ghonaim, Ahmad Almallah, Ahmed Fouad, Eman Adel Sayma, Ahmad Elbatahgy, Angham Solaiman El-Ma’doul, Ahmed Mosad, Hager Tolba, Diaa Eldin Abdelazeem Amin Elsorogy, Hassan Ali Mostafa, Amira Atef Omar, Ola Sherief Abd El Hameed, Ahmed Lasheen, Yasser Abd El Salam, Ashraf Morsi, Mohammed Ismail, Hager Ahmed El-badawy, Mohamed A. Amer, Ahmed Sabry El-Hamouly, Noura A. Attallah, Omnia Mosalum, Ahmed Afandy, Ahmed Mokhtar, Alaa Abouelnasr, Sara Ayad, Ramdan Shaker, Rokia Sakr, Ramadan Shaker, Mahmoud Amreia, Soaad Elsobky, Mohamed Mustafa, Ahmed Abo El Magd, Abeer Marey, Amr Tarek Hafez, Mohamed F. Zalabia, Mohamed Moamen Mohamed, Amr Fadel, Emad Ali Ahmed, Ahmad Ali, Mohammad Ghassan Alwafai, Abdullah Dwydar, Sara Kharsa, Ehab Mamdouh, Hatem El-Sheemy, Ibrahim AlYoussef, Abouelatta Khairy Aly, Ahmad Aldalaq, Ehab Alnawam, Dalia Alkhabbaz, Mahmoud Saad, Shady Hussein, Ahmed Abo Elazayem, Ahmed Meshref, Marwa Elashmawy, Mohammed Mousa, Ahmad Nashaat, Sara Ghanem, Zaynab M. Elsayed, Aya Elwaey, Iman Elkadsh, Mariam Darweesh, Ahmed Mohameden, Mennaallah Hafez, Ahmed Badr, Assmaa Badwy, Mohamed Abd El Slam, Mohamed Elazoul, Safwat Al-Nahrawi, Lotfy Eldamaty, Fathee Nada, Mohamed Ameen, Aya Hagar, Mohamed Elsehimy, Mohammad Aboraya, Hossam Dawoud, Shorouk El Mesery, Abeer El Gendy, Ahmed Abdelkareem, Ahmed Safwan Marey, Mostafa Allam, Sherif Shehata, Khaled Abozeid, Marwa Elshobary, Ahmed Fahiem, Sameh Sarsik, Amel Hashish, Mohamed Zidan, Mohamed Hashish, Shaimaa Aql, Abdelaziz Osman Abdelaziz Elhendawy, Mohamed Husseini, Esraa Kasem, Ahmed Gheith, Yasmin Elfouly, Ahmed Ragab Soliman, Yasmein Ibrahim, Nesma Elfouly, Ahmed Fawzy, Ahmed Hassan, Mohammad Rashid, Abdallah Salah Elsherbiny, Basem Sieda, Nermin M. Badwi, Mohammed Mustafa Hassan Mohammed, Osama Mohamed, Mohammad Abdulkhalek Habeeb, Mengistu Worku, Nichole Starr, Semay Desta, Sahlu Wondimu, Nebyou Seyoum Abebe, Efeson Thomas, Frehun Ayele Asele, Daniel Dabessa, Nebiyou Seyoum Abebe, Abebe Bekele Zerihun, Panu Mentula, Ari Leppäniemi, Ville Sallinen, Aurelien Scalabre, Fernanda Frade, Sabine Irtan, Vivien Graffeille, Elodie Gaignard, Quentin Alimi, Vivien Graffieille, Olivier Abbo, Sofia Mouttalib, Ourdia Bouali, Erik Hervieux, Yves Aigrain, Nathalie Botto, Alice Faure, Lucile Fievet, Nicoleta Panait, Emilie Eyssartier, Francoise Schmitt, Guillaume Podevin, Valentine Parent, Amandine Martin, Alexis Pierre Arnaud, Cecile Muller, Arnaud Bonnard, Matthieu Peycelon, Francis Abantanga, Kwaku Boakye-Yiadom, Mohammed Bukari, Frank Owusu, Joseph Awuku-Asabre, Lemuel Davies Bray, Dimitrios Lytras, Kyriakos Psarianos, Anastasia Bamicha, Eirini Kefalidi, Georgios Gemenetzis, Christos Dervenis, Nikolaos Gouvas, Christos Agalianos, Michail Kontos, Gregory Kouraklis, Dimitrios Karousos, Stylianos Germanos, Constantinos Marinos, Christos Anthoulakis, Nikolaos Nikoloudis, Nikolaos Mitroudis, Sergio Estupinian, Walter Forno, José René Arévalo Azmitia, Carla Cecilia Ramã-rez Cabrera, Romeo Guevara, Maria Aguilera, Napoleon Mendez, Cesar Augusto Azmitia Mendizabal, Pablo Ramazzini, Mario Contreras Urquizu, Fernando Tale, Rafael Soley, Emanuel Barrios, Emmanuel Barrios, Daniel Estuardo Marroquín Rodríguez, Carlos Iván Pérez Velásquez, Sara María Contreras Mérida, Francisco Regalado, Mario Lopez, Miguel Siguantay, Fong Yee Lam, Kylie Joan-yi Szeto, Charing Cheuk Ling Szeto, Wing Sum Li, Kieran Ka Kei Li, Man Fung Leung, Tony Mak, Simon Ng, S. S. Prasad, Anand Kirishnan, Nidhi Gyanchandani, Bylapudi Seshu Kumar, Muthukumaran Rangarajan, Sriram Bhat, Anjana Sreedharan, S. V. Kinnera, Yella Reddy, Caranj Venugopal, Sunil Kumar, Abhishek Mittal, Shravan Nadkarni, Harish Neelamraju Lakshmi, Puneet Malik, Neel Limaye, Srinivas Pai, Pratik Jain, Monty Khajanchi, Savni Satoskar, Rajeev Satoskar, Abid Bin Mahamood, Eldaa Prisca Refianti Sutanto, Daniel Ardian Soeselo, Chintya Tedjaatmadja, Fitriana Nur Rahmawati, Maria Mayasari, Ruqaya Kadhim Mohammed Jawad Al-Hasani, Hasan Ismael Ibraheem Al-Hameedi, Hasan Ismael Ibraheem, Israa Abdullah Aziz Al-Azraqi, Lubna Sabeeh, Rahma Kamil, Muwaffaq Mezeil Telfah, Amoudtha Rasendran, Jacqueline Sheehan, Robert Kerley, Caoimhe Normile, Richard William Gilbert, Jiheon Song, Mohamed Dablouk, Linnea Mauro, Mohammed Osman Dablouk, Paul Kielty, Eleanor Marks, Simon Gosling, Michelle Mccarthy, Diya Mirghani, Syed Altaf Naqvi, Chee Siong Wong, Siyi Chung, Reuban D’cruz, Ronan Cahill, Simon George Gosling, Ciara Fahy, Diana Duarte Cadogan, Anna Powell, Richard Gilbert, Caroline Clifford, Aoife Driscoll, Stassen Paul, Chris Lee, Ross Bowe, William Hutch, Helen Mohan, Maeve O’neill, Kenneth Mealy, Piergiorgio Danelli, Andrea Bondurri, Anna Maffioli, Mario Pasini, Giacomo Pata, Stefano Roncali, Paolo Silvani, Michele Carlucci, Roberto Faccincani, Luigi Bonavina, Yuri Macchitella, Chiara Ceriani, Gregorio Tugnoli, Salomone Di Saverio, Khaled Khattab, Miguel Angel Paludi, Domenica Pata, Luigi Maria Cloro, Andrea Allegri, Luca Ansaloni, Federico Coccolini, Ezio Veronese, Luca Bortolasi, Alireza Hasheminia, Giacomo Nastri, Massimiliano Dal Canto, Stefano Cucumazzo, Angelo Benevento, Gaetano Tessera, Pier Paolo Grandinetti, Alessio Maniscalco, Giovanni Luca Lamanna, Luca Turati, Giovanni Sgroi, Emanuele Rausa, Roberta Villa, Michela Monteleone, David Merlini, Veronica Grassi, Roberto Cirocchi, Alban Cacurri, Hamza Waleed, Ahmed Diab, Fathi Elzowawi, Mantas Jokubauskas, Karolis Varkalys, Donatas Venskutonis, Robertas Pranevicius, Viktorija Ambrozeviciute, Simona Juciute, Austė Skardžiukaitė, Saulius Bradulskis, Linas Urbanavicius, Aiste Austraite, Romualdas Riauka, Zilvinas Dambrauskas, Paulius Karumnas, Zigmantas Urniezius, Reda Zilinskiene, Anele Rudzenskaite, Ausrine Usaityte, Margarita Montrimaite, Nerijus Kaselis, Andrius Strazdas, Kristijonas Jokubonis, Kornelija Maceviciute, Virgilijus Beisa, Tomas Poskus, Kestutis Strupas, Erikas Laugzemys, Andrej Kolosov, Valdemaras Jotautas, Ignas Rakita, Saulius Mikalauskas, Darius Kazanavicius, Rokas Rackauskas, Ritauras Rakauskas, Egle Preckailaite, Ross Coomber, Kenneth Johnson, Jennifer Nowers, Dineshwary Periasammy, Afizah Salleh, Andre Das, Reuben Goh Ern Tze, Milaksh Nirumal Kumar, Nik Azim Nik Abdullah, Nik Ritza Kosai, Mustafa Taher, Reynu Rajan, Hoong Yin Chong, Marija Agius, Elaine Borg, Maureen Bezzina, Roberta Bugeja, Martinique Vella-Baldacchino, Andrew Spina, Josephine Psaila, Helene Francois-Coridon, Cecilia Tolg, Jean-Francois Colombani, Carmina Diaz-Zorrilla, Antonio Ramos-De La Medina, Samantha Corro-Diaz Gonzalez, Mário Jacobe, Domingos Mapasse, Elizabeth Snyder, Ramadan Oumer, Mohammed Osman, Aminu Mohammad, Lofty-John Anyanwu, Abdulrahman Sheshe, Alaba Adesina, Olubukola Faturoti, Ogechukwu Taiwo, Muhammad Habib Ibrahim, Abdulrasheed A. Nasir, Siyaka Itopa Suleiman, Adewale Adeniyi, Opeoluwa Adesanya, Ademola Adebanjo, Roland Osuoji, Kazeem Atobatele, Ayokunle Ogunyemi, Omolara Williams, Mobolaji Oludara, Olabode Oshodi, Adesoji Ademuyiwa, AbdulRazzaq Oluwagbemiga Lawal, Felix Alakaloko, Olumide Elebute, Adedapo Osinowo, Christopher Bode, Abidemi Adesuyi, Adesoji Tade, Adeleke Adekoya, Collins Nwokoro, Omobolaji O. Ayandipo, Taiwo Akeem Lawal, Akinlabi E. Ajao, Samuel Sani Ali, Babatunde Odeyemi, Samson Olori, Ademola Popoola, Ademola Adeyeye, James Adeniran, William J. Lossius, Ingemar Havemann, Kenneth Thorsen, Jon Kristian Narvestad, Kjetil Soreide, Trude Beate Wold, Linn Nymo, Mohammed Elsiddig, Manzoor Dar, Kamran Faisal Bhopal, Zainab Iftikhar, Muhammad Mohsin Furqan, Bakhtiar Nighat, Masood Jawaid, Abdul Khalique, Ahsan Zil-E-Ali, Anam Rashid, Hasnain Abbas Dharamshi, Tahira Naqvi, Ahmad Faraz, Abdul Wahid Anwar, Tahir Muhammad Yaseen, Ghina Shamim Shamsi, Ghina Shamsi, Tahir Yaseen, Wahid Anwer, Horacio Paredes Decoud, Omar Aguilera, Ismael Isaac Zelada Alvarez, Juan Marcelo Delgado, Gustavo Miguel Machain Vega, Helmut Alfredo Segovia Lohse, Wendy Leslie Messa Aguilar, Jose Antonio Cabala Chiong, Ana Cecilia Manchego Bautista, Eduardo Huaman, Sergio Zegarra, Rony Camacho, Jose María Vergara Celis, Diego Alonso Romani Pozo, José Hamasaki, Edilberto Temoche, Jaime Herrera-Matta, Carla Pierina García Torres, Luis Miguel Alvarez Barreda, Ronald Renato Barrionuevo Ojeda, Octavio Garaycochea, Melanie Castro Mollo, Mitchelle Solange De Fã Tima Linares Delgado, Francisco Fujii, Susana Yrma Aranzabal Durand, Carlos Alejandro Arroyo Basto, Nelson Manuel Urbina Rojas, Sebastian Bernardo Shu Yip, Ana Lucia Contreras Vergara, Andrea Echevarria Rosas Moran, Giuliano Borda Luque, Manuel Rodriguez Castro, Ramon Alvarado Jaramillo, George Manrique Sila, Crislee Elizabeth Lopez, Mardelangel Zapata Ponze De Leon, Massiell Machaca, Ronald Coasaca Huaraya, Andy Arenas, Crislee López, Clara Milagros Herrera Puma, Wilfredo Pino, Christian Hinojosa, Melanie Zapata Ponze De Leon, Susan Limache, George Manrrique Sila, Layza-Alejandra Mercado Rodriguez, Renato Melo, Jose Costa-Maia, Nuno Muralha, Frederique Sauvat, Ionasc Dan, Mircea Hogea, Pandi Eduard, Razvan-Matei Bratu, Mircea Beuran, Ionut-Bogdan Diaconescu, Bogdan-Valeriu Martian, Florin-Mihail Iordache, Mihaela Vartic, Lucian Corneliu Vida, Liviu Iuliu Muntean, Aurel Sandu Mironescu, Vizir Jean Paul Nsengimana, Alice Niragire, Jean De La Croix Allen Ingabire, Eugene Niyirera, Nicola Zanini, Elio Jovine, Giovanni Landolfo, Ibrahim N. Alomar, Saleh A. Alnuqaydan, Abdulrahman M. Altwigry, Moayad Othman, Nohad Osman, Enas Alqahtani, Mohammed Alzahrani, Rifan Alyami, Emad Aljohani, Ibrahim Alhabli, Zaher Mikwar, Sultan Almuallem, Abrar Nawawi, Mohamad Bakhaidar, Ashraf A. Maghrabi, Mohammed Alsaggaf, Murad Aljiffry, Abdulmalik Altaf, Ahmad Khoja, Alaa Habeebullah, Nouf Akeel, Nashat Ghandora, Abdullah Almoflihi, Abdulmalik Huwait, Abeer Al-shammari, Mashael Al-Mousa, Masood Alghamdi, Walid Adham, Bandar Albeladi, Muayad Ahmed Alfarsi, Atif Mahdi, Saad Al Awwad, Thamer Nouh, Mazen Hassanain, Salman Aldhafeeri, Nawal Sadig, Osama Algohary, Mohannad Aledrisy, Ahmad Gudal, Ahmad Alrifaie, Mohammed AlRowais, Amani Althwainy, Alaa Shabkah, Uthman Alamoudi, Mawaddah Alrajraji, Basim Alghamdi, Saud Aljohani, Abdullah Daqeeq, Jubran J. Al-Faifi, Vicky Jennings, Nyawira Ngayu, Rachel Moore, Victor Kong, Hayden Kretzmann, Katie Connor, Daniel Nel, Colleen Sampson, Eugenio Panieri, Nosisa Sishuba, Myint Tun, Albert Mohale Mphatsoe, Jo-Anne Carreira, Ella Teasdale, Mark Wagener, Stefan Botes, Danelo Du Plessis, Fernando Fernandez-Bueno, Jose Aguilar-Jimenez, Jose Andres Garcia-Marin, Lorena Solar García, Luis Joaquín García Florez, Rubén Darío Arias Pacheco, Janet Pagnozzi, Jimy Harold Jara Quezada, Jose Luis Rodicio, German Minguez, Raquel Rodríguez-Uría, Paul Ugalde, Camilo Lopez-Arevalo, Luis Barneo, Jessica Patricia Gonzales Stuva, Irene Ortega-Vazquez, Lorena Rodriguez, Norberto Herrera, Prasad Pitigala Arachchi, Wanigasekara Senanayake Mudiyanselage Kithsiri Janakantha Senanayake, Lalith Asanka Jayasooriya Jayasooriya Arachchige, Sivasuriya Sivaganesh, Dulan Irusha Samaraweera, Vimalakanthan Thanusan, Ahmed Elgaili Khalid Musa, Reem Mohammed Hassan Balila, Mohamed Awad Elkarim Hamad Mohamed, Hussein Ali, Hagir Zain Elabdin, Alaa Hassan, Sefeldin Mahdi, Hala Ahmed, Sahar Abdoun Ishag Idris, Makki Elsayed, Mohammed Elsayed, Mohamed Mahmoud, Magnus Boijsen, Per-Olof Lundgren, Ulf Gustafsson, Ali Kiasat, Fredrik Wogensen, Emma Jurdell, Anders Thorell, Hildur Thorarinsdottir, Maria Utter, Sami Martin Sundstrom, Cecilia Wredberg, Ann Kjellin, Johanna Nyberg, Bjorn Frisk, Malin Sund, Linda Andersson, Ulf Gunnarsson, Yücel Cengiz, Sandra Ahlqvist, Ida Björklund, Hanna Royson, Per Weber, Hans-Ivar Pahlsson, Eva Borin, Maria Hjertberg, Roger Schmid, Debora Schivo, Vasileios Despotidis, Stefan Breitenstein, Ralph F. Staerkle, Erik Schadde, Fabian Deichsel, Alexandra Gerosa, Antonio Nocito, Dimitri Aristotle Raptis, Barbara Mijuskovic, Markus Zuber, Lukas Eisner, Swantje Kruspi, Katharina Beate Reinisch, Christin Schoewe, Allan Novak, Adrian F. Palma, Gerfried Teufelberger, Msafiri Kimaro, Rachel King, Ali Zeynel Abidin Balkan, Mehmet Gumar, Mehmet Ali Yavuz, Ufuk Karabacak, Gokhan Lap, Bahar Busra Ozkan, Murat Karakahya, Ryan Adams, Robert Morton, Liam Henderson, Ruth Gratton, Keiran David Clement, Kate Yu-Ching Chang, David Mcnish, Ryan Mcintosh, William Milligan, Brendan Skelly, Hannah Anderson-Knight, Roger Lawther, Jemina Onimowo, Veereanna Shatkar, Shivanee Tharmalingam, Evelina Woin, Tessa Fautz, Oliver Ziff, Shiva Dindyal, Sam Arman, Shagorika Talukder, Vijay Gadhvi, Luen Shaun Chew, Jonathan Heath, Natalie Blencowe, Sally Hallam, Katherine Gash, Gurdeep Singh Mannu, Dimitris-Christos Zachariades, Ailsa Claire Snaith, Thusitha Sampath Hettiarachchi, Arjun Nesaratnam, James Wheeler, Darragh McCullagh, Joshua Michael Clements, Ata Khan, Foteini Koumpa, Christina Neophytou, Jessica Roth, Wai Cheong Soon, Mohammed Deputy, Ahmed Ahmed, Annelisse Ashton, Joe Vincent, Jack Almy, Taufiq Khan, John Lee Y. Allen, Charlotte Jane Mcintyre, Dominic Charles Marshall, Mark Sykes, Nebil Behar, Harriet Jordan, Yaseen Rajjoub, Thomas Sherman, Timothy White, Anna Watts, Rohan Ardley, Tan Arulampalam, Apar Shah, Damien Brown, Emma Blower, Paul Sutton, Konstantinos Gasteratos, Dale Vimalachandran, Cathy Magee, Andrew Mcguigan, Stephen Mcaleer, Clare Morgan, Sarah Braungart, Kirsten Lafferty, Peter Labib, Andrei Tanase, Clodagh Mangan, Lillian Reza, Helen Woodward, Craig Gouldthorpe, Megan Turner, Jonathan R. L. Wild, Tom A. M. Malik, Victoria K. Proctor, Kalon Hewage, James Davies, Andre Dubois, Sayed Sarwary, Ali Zardab, Alan Grant, Robert Mcintyre, Yogendra Praveen Mogan, Weiguang Ho, Bryon Frankie Hon Khi Chong, Shirish Tewari, Gemma Humm, Eriberto Farinella, Nigel J. Hall, Naomi J. Wright, Christina P. Major, Thelma Xerri, Jasim Amin, Mustafa Farhad, John F. Camilleri-Brennan, Andrew G. N. Robertson, Joanna Swann, James Richards, Aijaz Jabbar, Phoebe De Bono, Myranda Attard, Hannah Burns, Euan Macdonald, Matthew Baldacchino, Jennifer Skehan, Tom Falconer Hall, Madelaine Gimzewska, Greta Mclachlan, Jamie Shah, James Giles Selina Chiu, Beatrix Weber, Selina Man Yeng Chiu, Saskia Highcock, Maleeha Hassan, William Beasley, Apostolos Vlachogiorgos, Stephen Dias, Geta Maharaj, Rosie Mcdonald, Alisdair Macdonald, Paul Witherspoon, Alan Baird, Panchali Sarmah, Nikki Green, Haney Youssef, Kate Cross, Clare M. Rees, Bernard Van Duren, Emma Upchurch, Khurram Khan, Haytham Abudeeb, Ahmed Hammad, Sharad Karandikar, Doug Bowley, Ahmed Karim, Witold Chachulski, Liam Richardson, Giles Dawnay, Ben Thompson, Ajayesh Mistry, Millika Ghetia, Sudipta Roy, Ossama Al-Obaedi, Kaustuv Das, Ash Prabhudesai, D. M. Cocker, Jessica Juliana Tan, Robert Tyler, Filippo Di Franco, Shruti Ayyar, Sayinthen Vivekanantham, Shyam Gokani, Michael Gillespie, Katrin Gudlaugsdottir, Theodore Pezas, Chelise Currow, Matthew Young-Han Kim, Amerdip Birring, Joanne Edwards, Ased Ali, Suparna Das, Madan Jha, Kieran Atkinson, Joshua Luck, Thomas Fozard, Michael Puttick, Yahya Salama, Rohi Shah, Ahmad Aboelkassem Ibrahem, Hamdi Ebdewi, Gianpiero Gravante, Saleem El-Rabaa, Henry Nnajiuba, Rebecca Allott, Aman Bhargava, Zoe Chan, Zaffar Hassan, Misty Makinde, David Hemingway, Ramzana Dean, Alexander Boddy, Ahmed Aber, Vijay Patel, Jehangirshaw Parakh, Sunil Parthiban, Harmony Kaur Ubhi, Simon-Peter Hosein, Simon Ward, Kamran Malik, Leifa Jennings, Tom Newton, Mirna Alkhouri, Min Kyu Kang, Christopher Houlden, Jonathan Barry, Imtanan Raza, Alistair Farquharson, Sanjeet Bhattacharya, Kate Chang, Michael S. J. Wilson, Yan Ning Neo, Ibrahim Ibrahim, Emily Chan, Fraser S. Peck, Pei J. Lim, Alexander S. North, Rebecca Blundell, Adam Williamson, Dina Fouad, Ashish Minocha, Kathryn Mccarthy, Emma Court, Alice Chambers, Jenna Yee, Ji Chung Tham, Ceri Beaton, Una Walsh, Joseph Lockey, Salman Bokhari, Lara Howells, Megan Griffiths, Laura Yallop, Shailinder Singh, Omar Nasher, Paul Jackson, Abdul Muiz Shariffuddin, Weng Chee Ho, Michael Sj Wilson, Gurpreet Pabla, Saed Ramzi, Shady Zeidan, Jennifer Doughty, Sidhartha Sinha, Ross Davenport, Jason Lewis, Leo Duffy, Elizabeth Mcaleer, Eleanor Williams, Robin Som, Omar Javed, Matthew Boal, Nicola Harrison, Habib Tafazal, Tom Brogden, Ewen Griffiths, Rhalumi Daniel Obute, Thomas E. Glover, David J. Clark, Mohamed Boshnaq, Mansoor Akhtar, Pascale Capleton, Samer Doughan, Mohamed Rabie, Ismail Mohamed, Duncan Samuel, Lauren Dickson, Matthew Kennedy, Eleanor Dempster, Emma Brown, Natalie Maple, Eimear Monaghan, Bernhard Wolf, Alicia Garland, Arthur Mcphee, David Anderson, Robert Anderson, Sarah Hassan, Dave Smith, Jonathan Lund, Catherine Boereboom, Jennifer Murphy, Gillian Tierney, Samson Tou, Eleanor Franziska Zimmermann, Neil James Smart, Andrea Marie Warwick, Theodora Stasinou, Ian Daniels, Kim Findlay-Cooper, Stefan Mitrasinovic, Swayamjyoti Ray, Massimo Varcada, Rovan D’Souza, Sharif Omara, Matthew Spurr, Lucienne Parkinson, Anthony Hanks, Jennifer Ma, Emily Abington, Meera Ramcharn, Gethin Williams, Joseph Winstanley, Ewan D. Kennedy, Emily N. W. Yeung, Catrin Jones, Stephen O’neill, Shujing Jane Lim, Ignatius Liew, Hari Nair, Cameron Fairfield, Julia Oh, Samantha Koh, Andrew Wilson, Catherine Fairfield, Delran Anandkumar, Ashok Kirupagaran, Timothy F. Jones, Hew Dt Torrance, Alexander J. Fowler, Charmilie Chandrakumar, Priyank Patel, Syed Faaz Ashraf, Sonam M. Lakhani, Aaron Lawson Mclean, Sonia Basson, Jeremy Batt, Catriona Bowman, Michael Stoddart, Natasha Benons, Clare Mason, Rebecca Harrison, John Quayle, Tom Barker, Virginia Summerour, Edward Harper, Caroline Smith, Matthew Hampton, Sophie K. Pitt, Alex E. Ward, Timothy O’Connor, Emily G. Heywood, Abeed Chowdhury, Sina Hossaini, Nicholas Fs Watson, Doug Mckechnie, Ayaan Farah, Anita Chun, Hoey Koh, Grace Lim, Graham Sunderland, Laura Gould, P. C. Munipalle, H. Rooney, D. R. L. Browning, Bernadette Pereira, Kristof Nemeth, Emily Decker, Stefano Giuliani, Aly Shalaby, Shafaque Shaikh, Chern Yan Tan, Ebrahim Y. A. Palkhi, Aleksandra Szczap, Swathikan Chidambaram, Chee Yang Chen, Kavian Kulasabanathan, Srishti Chhabra, Elisabeth Kostov, Philippe Harbord, James Barnacle, Madan Mohan Palliyil, Mina Zikry, Johnathan Porter, Charef Raslan, Shazia Hafiz, Niksa Soltani, Katie Baillie, Priyanka Singh, Shailee Sheth, Kishen Patel, Mahry Khalili, Jeesoo Choi, Matthew Benger, Lucy Marples, Alastair Macfarlane, Ramesh Thurairaja, Tamsin Boyce, Harriet Whewell, Elin Jones, Francesca Th’ng, Nichola Robertson, Ahmad Mirza, Haroon Saeed, Simon Galloway, Gia Elena, Mohammad Afzal, Mohamed Zakir, Peter Sodde, Charles Hand, Aiesha Sriram, Tamsyn Clark, Patrick Holton, Amy Livesey, Yashashwi Sinha, Fahad Mujtaba Iqbal, Indervir Singh Bharj, Adriana Rotundo, Cara Jenvey, Robert Slade, David Golding, Samuel Haines, Ali Adel Ne’ma Abdullah, Thomas W. Tilston, Dafydd Loughran, Danielle Donoghue, Lorenzo Giacci, Mohamed Ashur Sherif, Peter Harrison, Alethea Tang, Deevia Kotecha, Mohamed Elshaer, Tomas Urbonas, Amjid Riaz, Annie Chapman, Parisha Acharya, Joseph Shalhoub, Cathleen Grossart, David McMorran, Makhosini Mlotshwa, William Hawkins, Sofronis Loizides, Kandaswamy Krishna, Melanie Orchard, Chik Wai Ho, Peter Thomson, Shahab Khan, Fiona Taylor, Jalak Shukla, Emma Elizabeth Howie, Linda Macdonald, Olusegun Komolafe, Neil Mcintyre, James Cragg, Jody Parker, Duncan Stewart, Luke Lintin, Julia Tracy, Tahir Farooq, George Molina, Haytham Kaafarani, Robel Beyene, Jack Sava, Mark Scott, Mamta Swaroop, Raelene Kennedy, Ijeoma A. Azodo, Daithi Heffernan, Tristen Chun, Andrew Stephen, Melanie Sion, Michael S. Weinstein, Viren Punja, Nikolay Bugaev, Monica Goodstein, Shadi Razmdjou, Eric Etchill, Juan Carlos Puyana, Matthew Kesinger, Lena Napolitano, Kathleen To, Mark Hemmila, Oliver Todd, Edward Jenner, Ellen Hoogakker, Jacky Hong Chieh Chen, Lawani Ismail, Dylan Roi, Eugenio Grasset Escobar

**Affiliations:** NIHR Unit on Global Surgery (Universities of Birmingham, Edinburgh and Warwick), University of Edinburgh, Royal Infirmary of Edinburgh, Edinburgh, EH16 4SA UK

**Keywords:** Appendicitis, Appendectomy, Global surgery, Laparoscopic, Operative standards, Postoperative care, Postoperative complications, Surgical site infection

## Abstract

**Background:**

Appendicitis is the most common abdominal surgical emergency worldwide. Differences between high- and low-income settings in the availability of laparoscopic appendectomy, alternative management choices, and outcomes are poorly described. The aim was to identify variation in surgical management and outcomes of appendicitis within low-, middle-, and high-Human Development Index (HDI) countries worldwide.

**Methods:**

This is a multicenter, international prospective cohort study. Consecutive sampling of patients undergoing emergency appendectomy over 6 months was conducted. Follow-up lasted 30 days.

**Results:**

4546 patients from 52 countries underwent appendectomy (2499 high-, 1540 middle-, and 507 low-HDI groups). Surgical site infection (SSI) rates were higher in low-HDI (OR 2.57, 95% CI 1.33–4.99, *p* = 0.005) but not middle-HDI countries (OR 1.38, 95% CI 0.76–2.52, *p* = 0.291), compared with high-HDI countries after adjustment. A laparoscopic approach was common in high-HDI countries (1693/2499, 67.7%), but infrequent in low-HDI (41/507, 8.1%) and middle-HDI (132/1540, 8.6%) groups. After accounting for case-mix, laparoscopy was still associated with fewer overall complications (OR 0.55, 95% CI 0.42–0.71, *p* < 0.001) and SSIs (OR 0.22, 95% CI 0.14–0.33, *p* < 0.001). In propensity-score matched groups within low-/middle-HDI countries, laparoscopy was still associated with fewer overall complications (OR 0.23 95% CI 0.11–0.44) and SSI (OR 0.21 95% CI 0.09–0.45).

**Conclusion:**

A laparoscopic approach is associated with better outcomes and availability appears to differ by country HDI. Despite the profound clinical, operational, and financial barriers to its widespread introduction, laparoscopy could significantly improve outcomes for patients in low-resource environments.

Trial registration: NCT02179112.

**Electronic supplementary material:**

The online version of this article (10.1007/s00464-018-6064-9) contains supplementary material, which is available to authorized users.

The global burden of diseases requiring emergency surgery is poorly described and represents a significant health problem [1]. Most of the world’s population do not have access to safe, affordable and timely surgery, with access inequitably distributed in favor of high Human Development Index (HDI) countries [2]. It is estimated that in 2010, approximately 17 million deaths resulted from conditions requiring surgical care, far greater than the combined burden of HIV/AIDs, malaria, and tuberculosis [3]. There is an urgent need to increase access to surgical treatment across the world.

Appendicitis is one of the commonest diseases requiring emergency abdominal surgery, yet little data exist to allow comparison of management and outcomes at a patient level globally [4]. Recently published trends predict a significant increase in the prevalence of appendicitis in newly industrialized countries [5]. Data from the Global Burden of Disease Study (2016) show a higher prevalence of appendicitis in lower socio-economic countries, together with a greater proportion of years-of-life-lost as a result of the disease [6]. Appendicitis is usually treated with surgery, although management purely with antibiotics has been investigated [7]. Appendectomy can be performed by a traditional open procedure, but a laparoscopic approach has become common in many countries [8]. Significant variation in practice still exists in high-income settings [9].

The role of laparoscopy in low-resource healthcare settings has been debated [10]. Those arguing against its use suggest that the required initial financial and training investment, together with the on-going costs of equipment upkeep and consumables, make it unviable when compared to relatively straightforward open surgery. This argument has resonance: when a healthcare system is struggling to deliver basic surgical procedures, the introduction of a more complex intervention must be considered carefully. On the other hand, there are broad advantages to having laparoscopy available, particularly the ability to perform diagnostic laparoscopy in the absence of expensive CT imaging. Lower postoperative complication rates [9] and consequent healthcare costs are commonly reported to be associated with laparoscopic appendectomy. Whether laparoscopy has the same advantage in low-resource settings is unknown.

The GlobalSurg Collaborative has recently demonstrated the feasibility of conducting international data collection in low-resource settings [4, 11]. Using these approaches, this study aimed to investigate the surgical management of appendectomy worldwide, including the use of laparoscopy, and to examine outcomes following surgery.

## Methods

### Study setting

A collaborative, international, multicenter, prospective, observational cohort study was conducted according to a pre-specified, published protocol (ClinicalTrials.gov identifier: NCT02179112) [12]. The collaborative network methodology has been described elsewhere [13]. Briefly, the study was conducted by teams of local investigators coordinated by a national lead investigator. Investigators were recruited via the GlobalSurg network and through dissemination on social media and other personal contacts. Consecutive sampling of patients undergoing emergency abdominal surgery was undertaken during 2-week periods within a 6-month study window. Investigators in a hospital could choose one or multiple 2-week periods. There was an absolute requirement for all cases in the chosen period(s) to be included, but no minimum number set to avoid bias against smaller centers. A UK National Health Service Research Ethics review considered this study exempt from formal research registration (South East Scotland Research Ethics Service, reference NR/1404AB12); individual centers obtained their own audit, ethical or institutional approval. This study is reported according to the Strengthening the Reporting of Observational Studies in Epidemiology (STROBE) guidelines [14]. Pre-specified sub-group analyses included the examination of patients undergoing appendectomy as compared with other abdominal surgical procedures. At each participating center, data collection was performed by teams following the same standardized protocol [12].

### Patients and procedures

The study inclusion period was 01 July 2014 to 31 December 2014. Consecutive patients undergoing emergency appendectomy were eligible for inclusion. No limits were placed on age or operative approach. Emergency surgery was defined as any unplanned, non-elective operation, including re-operation after a previous procedure. Elective (planned) or semi-elective procedures (where a patient initially admitted as an emergency was then discharged from hospital and re-admitted at later time for surgery) were excluded.

### Data

Data were selected to be objective, standardized, easily transcribed and internationally relevant, to maximize record completion and accuracy. Patients were followed for 30 days after surgery or for the length of their inpatient stay where follow-up was not feasible. Records were uploaded by local investigators to a secure online website, provided using the Research Electronic Data Capture (REDCap) system [15]. The lead investigator at each site was responsible for “signing off” patient records. Data were checked with primary data sources if necessary. The local lead was responsible for ensuring consecutive sampling (i.e., no excluded cases) for each site. The submitted data were then checked centrally and where missing data were identified, the local lead investigator was contacted and asked to investigate. Once vetted, the record was accepted into the dataset for analysis.

Patient variables included age, gender, American Society of Anesthesiologists (ASA) physical status classification system, diabetes history, smoking and diagnostic category. Service variables included use of the WHO surgical safety checklist, the experience of surgeon/anesthetist, and compliance with selected evidence-based standards. Prophylactic antibiotic use was defined as antibiotics administered either at induction, or during surgery but before opening of a contaminated space.

### Outcome variables

The primary outcome measure was overall complication rate, defined using the Clavien–Dindo grade occurring within 30 days of the index operation. The criteria required for each outcome were defined a priori in the protocol [12]. Major complication (Clavien–Dindo IV) was defined as a life-threatening illness requiring critical care management. In low-resource settings, investigators could report “complication requiring critical care, but facilities unavailable.”

The US Centre for Disease Control and Prevention (CDC) definitions for SSI and organ space infection (OSI) were used [16]. SSI: one of (1) purulent drainage from the incision; (2) at least two of following: pain or tenderness, localized swelling, redness, heat, fever, and the incision is opened deliberately to manage infection; (3) wound organisms AND pus cells from aspirate/swab. OSI: intra-abdominal/pelvic detected clinically/symptomatically, radiologically, or intra-operatively.

### Power considerations

The sample size was limited by practical factors and estimation of power by uncertainty over critical quantities such as clustering and variation by diagnosis. An indicative calculation for overall complication rate showed that considerable precision was likely with 520 patients per HDI comparison group (a 6% point difference in overall complication rate; baseline rate 16% with alpha = 0.05, beta = 0.2, and accounting for missing data/loss to follow-up).

### Statistical analysis

Variation across different international health settings was assessed by stratifying participating centers by country into three tertiles according to HDI rank. This is a composite statistic of life expectancy, education, and income indices published by the United Nations (http://hdr.undp.org/en/statistics). Differences between HDI tertiles were initially tested with the Pearson chi-squared and Kruskal–Wallis tests for categorical and continuous variables respectively.

Multivariable binary logistic regression models were constructed to adjust for case-mix and measures of hospital facilities and service, to explore the nature of differences in outcome. Subsequently, three-level hierarchical multivariable logistic regression models were made to account for hospital- and country-level variation (“hospital” and “country” as random effects with constrained gradients). Variable selection was incremental and accounted for both clinical and statistical significances. Model fit was guided log-likelihood methods including the Akaike Information Criterion (AIC). Discrimination was defined by the area-under-the receiver-operator-curve (c-statistic). Calibration across the range of observed probabilities was checked. All two-way interactions were investigated. Bayesian simulation methods are described in the supplementary digital content.

In a further propensity-score matched study, patients from low- and middle-HDI countries were analyzed separately to those from high-HDI countries. Patients who had undergone laparoscopic surgery were matched with those who underwent open surgery using a “nearest neighbor” approach, based on probabilities of group membership determined with multivariable logistic regression. The matching algorithm used age, gender, smoking status, ASA score, and perforation status. Pre- and post-matching balance was checked for all relevant variables.

All analyses were undertaken using the R Foundation Statistical Program (R 3.1.1, R Foundation for Statistical Computing) and Stan (Stan: A C++ Library for Probability and Sampling, Version 2.10.0. URL http://mc-stan.org/).

## Results

4546 patients underwent laparoscopic or open appendectomy for acute appendicitis over the data collection period.

### Demographics

Patients were from 52 countries with high-HDI (*n* = 2499), middle-HDI (*n* = 1540), and low-HDI (*n* = 507) groups (Table 1). Patients in low-HDI countries were younger (Pearson’s χ^2^ test, *p* < 0.001) and more likely to be male [low-HDI (295/507, 58%), middle-HDI (783/1540, 51%), high-HDI (1344/2499, 54%), *p* = 0.012]. There were numerically more patients considered to have severe systemic disease (ASA 3 or greater) in the low-HDI group (*p* = 0.021).

**Table 1:** Patient and operative characteristics

	Human development index (HDI)			*p* Value
	High (*n* = 2499)	Middle (*n* = 1540)	Low (*n* = 507)	
Age in completed years				
Mean (SD)	31.2 (18.2)	26.9 (14.6)	24.1 (12.1)	< 0.001
Gender				
Male	1344 (53.8)	783 (50.8)	295 (58.2)	0.012
Female	1155 (46.2)	757 (49.2)	212 (41.8)	
Diabetes history				
No	2435 (97.4)	1493 (96.9)	489 (96.4)	0.390
Yes	64 (2.6)	47 (3.1)	18 (3.6)	
Smoking currently				
No	1977 (79.1)	1295 (84.1)	446 (88.0)	< 0.001
Yes	521 (20.8)	244 (15.8)	60 (11.8)	
Missing	1 (0.0)	1 (0.1)	1 (0.2)	
ASA score				
1	1724 (69.0)	1098 (71.3)	344 (67.9)	0.021
2	613 (24.5)	332 (21.6)	102 (20.1)	
≥ 3	104 (4.2)	74 (4.8)	33 (6.5)	
Missing	58 (2.3)	36 (2.3)	28 (5.5)	
Procedure start time				
0800–1800 (daytime)	1345 (53.8)	615 (39.9)	188 (37.1)	< 0.001
1800–2200 (evening)	616 (24.6)	412 (26.8)	137 (27.0)	
2200–0800 (night-time)	538 (21.5)	513 (33.3)	182 (35.9)	
Surgical safety checklist used				
No, not available in this hospital	182 (7.3)	570 (37.0)	187 (36.9)	< 0.001
No, but available in this hospital	35 (1.4)	103 (6.7)	161 (31.8)	
Yes	2282 (91.3)	867 (56.3)	159 (31.4)	
Prophylactic antibiotics				
No	223 (8.9)	205 (13.3)	62 (12.2)	< 0.001
Yes	2276 (91.1)	1335 (86.7)	445 (87.8)	< 0.001
Senior surgeon > 5 years training				
No	108 (4.3)	769 (49.9)	210 (41.4)	< 0.001
Yes	2391 (95.7)	770 (50.0)	297 (58.6)	
Missing	0 (0.0)	1 (0.1)	0 (0.0)	
Senior anesthetist > 5 years training				
No	115 (4.6)	789 (51.2)	265 (52.3)	< 0.001
Yes	2384 (95.4)	751 (48.8)	242 (47.7)	
Laparoscopic approach				
No	806 (32.3)	1408 (91.4)	466 (91.9)	< 0.001
Yes	1693 (67.7)	132 (8.6)	41 (8.1)	
Perforated viscus				
No	2150 (86.0)	1380 (89.6)	394 (77.7)	< 0.001
Yes	348 (13.9)	159 (10.3)	109 (21.5)	
Missing	1 (0.0)	1 (0.1)	4 (0.8)	

Data are *n* (%) unless otherwise stated

*SD* standard deviation

### Surgical characteristics

There were clear differences in management between HDI groups (Table 1). A laparoscopic approach was used in 67.7% of patients in the high-HDI group and in < 10% in the low- and middle-HDI groups (Pearson’s χ^2^ test, *p* < 0.001). There was more operating through the night in low- and middle-HDI groups (Pearson’s χ^2^ test, *p* < 0.001), while more patients were operated upon > 24 h after admission in high-HDI countries. Seniority of both the surgeon and anesthetist was higher in high-HDI countries. Prophylactic antibiotics and use of the WHO surgical safety checklist was less common in low- and middle-HDI groups.

### Outcomes

In univariable analyses, associations were seen between HDI and overall and minor complications, as well as surgical site and OSIs (Table 2). There was no strong association between HDI and reintervention, major complication, or death.

**Table 2:** Outcomes

	Human development index (HDI)			
	High (*n* = 2499)	Middle (*n* = 1540)	Low (*n* = 507)	*p* Value
Overall complications (Clavien–Dindo I, II, III, IV, or V)				
No	2182 (87.3)	1303 (84.7)	414 (81.7)	0.001
Yes	317 (12.7)	235 (15.3)	93 (18.3)	
Missing	0 (0.0)	2 (0.1)	0 (0.0)	
Minor complication (Clavien–Dindo I/II)				
No	2219 (88.9)	1314 (86.1)	423 (84.1)	0.002
Yes	278 (11.1)	212 (13.9)	80 (15.9)	
Missing	2 (0.1)	14 (0.9)	4 (0.8)	
Reintervention (Clavien–Dindo III)				
No	2431 (97.3)	1501 (97.6)	494 (97.4)	0.828
Yes	68 (2.7)	37 (2.4)	13 (2.6)	
Missing	0 (0.0)	2 (0.1)	0 (0.0)	
Major complication (Clavien–Dindo IV)				
No	2474 (99.0)	1520 (98.8)	498 (98.2)	0.325
Yes	25 (1.0)	18 (1.2)	9 (1.8)	
Missing	0 (0.0)	2 (0.1)	0 (0.0)	
Surgical site infection (SSI)				
No	2389 (95.6)	1340 (88.0)	432 (85.2)	< 0.001
Yes	109 (4.4)	183 (12.0)	75 (14.8)	
Missing	1 (0.0)	1 (0.1)	0 (0.0)	
Organ/space infection (OSI)				
No	2397 (96.0)	1486 (96.4)	494 (97.4)	0.023
Yes	101 (4.0)	39 (2.7)	13 (2.6)	
Missing	1 (0.0)	15 (1.0)	0 (0.0)	
Length of stay after surgery (days)				
Mean (SD)	3 (3.4)	2.9 (3.2)	4.1 (4.7)	< 0.001
30-day mortality				
Alive	2496 (99.9)	1532 (99.5)	504 (99.4)	0.061
Dead	3 (0.1)	2 (0.1)	3 (0.6)	
Missing	0 (0.0)	6 (0.4)	0 (0.0)	

Data are *n* (%) unless otherwise stated

*SD* standard deviation

The overall complication rate was higher in low-HDI (odds ratio (OR) 1.55, 95% confidence interval (CI) 1.19–1.99, *p* = 0.001) and middle-HDI (OR 1.24, 95% CI 1.03–1.49, *p* = 0.020) countries compared with the high-HDI group (Tables 2, 3). When the analysis was adjusted in a multilevel model accounting for patient, disease, and hospital structural factors, there was no independent association between overall complications and HDI group.

**Table 3:** Overall complications

	Overall complications			
	No	Yes	Univariable logistic regression OR (95% CI, *p* value)	Multilevel logistic regression OR (95% CI, *p* value)
HDI tertile				
High	2182 (56.0)	317 (49.1)		
Middle	1303 (33.4)	235 (36.4)	1.24 (1.03–1.49, *p* = 0.020)	0.91 (0.56–1.48, *p* = 0.701)
Low	414 (10.6)	93 (14.4)	1.55 (1.19–1.99, *p* = 0.001)	1.44 (0.82–2.54, *p* = 0.209)
Age in completed years				
Mean (SD)	28.2 (16)	33.3 (19.5)	1.02 (1.01–1.02, *p* < 0.001)	1.01 (1.01–1.02, *p* < 0.001)
Gender				
Male	2069 (53.1)	351 (54.4)		
Female	1830 (46.9)	294 (45.6)	0.95 (0.80–1.12, *p* = 0.523)	0.98 (0.82–1.19, *p* = 0.873)
Diabetes history				
No	3805 (97.6)	610 (94.6)		
Yes	94 (2.4)	35 (5.4)	2.32 (1.54–3.42, *p* < 0.001)	1.27 (0.80–2.01, *p* = 0.315)
Smoking currently				
No	3208 (82.3)	508 (78.8)		
Yes	688 (17.7)	137 (21.2)	1.26 (1.02–1.54, *p* = 0.029)	0.96 (0.76–1.23, *p* = 0.770)
ASA score				
1	2790 (73.6)	375 (59.4)		
2	850 (22.4)	196 (31.1)	1.72 (1.42–2.07, *p* < 0.001)	1.42 (1.13–1.78, *p* = 0.003)
≥ 3	151 (4.0)	60 (9.5)	2.96 (2.14–4.04, *p* < 0.001)	1.64 (1.10–2.46, *p* = 0.016)
Procedure start time				
0800–1800 (daytime)	1854 (47.6)	292 (45.3)		
1800–2200 (evening)	985 (25.3)	180 (27.9)	1.16 (0.95–1.42, *p* = 0.148)	
2200–0800 (night-time)	1060 (27.2)	173 (26.8)	1.04 (0.85–1.27, *p* = 0.730)	
Surgical safety checklist used				
No, not available in this hospital	781 (20.0)	157 (24.3)		
No, but available in this hospital	250 (6.4)	48 (7.4)	0.96 (0.67–1.35, *p* = 0.799)	0.79 (0.48–1.30, *p* = 0.352)
Yes	2868 (73.6)	440 (68.2)	0.76 (0.63–0.93, *p* = 0.008)	0.84 (0.59–1.19, *p* = 0.327)
Prophylactic antibiotics				
No	422 (10.8)	67 (10.4)		
Yes	3477 (89.2)	578 (89.6)	1.05 (0.80–1.39, *p* = 0.741)	0.99 (0.72–1.37, *p* = 0.974)
Senior surgeon > 5 years training				
No	920 (23.6)	167 (25.9)		
Yes	2978 (76.4)	478 (74.1)	0.88 (0.73–1.07, *p* = 0.207)	1.05 (0.78–1.42, *p* = 0.745)
Senior anesthetist > 5 years training				
No	980 (25.1)	189 (29.3)		
Yes	2919 (74.9)	456 (70.7)	0.81 (0.67–0.98, *p* = 0.025)	1.02 (0.75–1.39, *p* = 0.901)
Laparoscopic approach				
No	2222 (57.0)	457 (70.9)		
Yes	1677 (43.0)	188 (29.1)	0.55 (0.45–0.65, *p* < 0.001)	0.55 (0.42–0.71, *p* < 0.001)
Perforated viscus				
No	3474 (89.2)	450 (69.8)		
Yes	419 (10.8)	195 (30.2)	3.59 (2.95–4.37, *p* < 0.001)	3.66 (2.91–4.62, *p* < 0.001)

Data are *n* (%) unless otherwise stated. Hospitals = 339, countries = 52. AIC = 3339.1. c-statistic = 0.790

*OR* odds ratio, *CI* confidence interval, *HDI* human development index, *ASA* American Association of Anesthesiologists risk score, *SD* standard deviation

A further prominent association was seen with more surgical site infection (SSI) in low-HDI (OR 3.81, 95% CI 2.78–5.19, *p* < 0.001) and middle-HDI (OR 2.99, 95% CI 2.34–3.84, *p* < 0.001) compared with high-HDI (Tables 2, 4). In the multilevel model, the association persisted in low-HDI (OR 2.57, 95% CI 1.33–4.99, *p* = 0.005) but not middle-HDI (OR 1.38, 95% CI 0.76–2.52, *p* = 0.291) countries.

**Table 4:** Surgical site infection

	Surgical site infection			
	No	Yes	Univariable logistic regression OR (95% CI, *p* value)	Multilevel logistic regression OR (95% CI, *p* value)
HDI tertile				
High	2389 (57.4)	109 (29.7)		
Middle	1340 (32.2)	183 (49.9)	2.99 (2.34–3.84, *p* < 0.001)	1.38 (0.76–2.52, *p* = 0.291)
Low	432 (10.4)	75 (20.4)	3.81 (2.78–5.19, *p* < 0.001)	2.57 (1.33–4.99, *p* = 0.005)
Age in completed years				
Mean (SD)	28.6 (16.4)	32.8 (18.9)	1.01 (1.01–1.02, *p* < 0.001)	1.01 (1.01–1.02, *p* = 0.001)
Gender				
Male	2205 (53.0)	208 (56.7)		
Female	1956 (47.0)	159 (43.3)	0.86 (0.69–1.07, *p* = 0.175)	0.95 (0.74–1.22, *p* = 0.666)
Diabetes history				
No	4058 (97.5)	341 (92.9)		
Yes	103 (2.5)	26 (7.1)	3.00 (1.89–4.61, *p* < 0.001)	1.45 (0.83–2.52, *p* = 0.189)
Smoking currently				
No	3415 (82.1)	287 (78.2)		
Yes	743 (17.9)	80 (21.8)	1.28 (0.98–1.65, *p* = 0.062)	1.05 (0.77–1.45, *p* = 0.751)
ASA score				
1	2937 (72.6)	213 (58.7)		
2	934 (23.1)	111 (30.6)	1.64 (1.28–2.08, *p* < 0.001)	1.42 (1.05–1.94, *p* = 0.025)
≥ 3	172 (4.3)	39 (10.7)	3.13 (2.13–4.50, *p* < 0.001)	1.82 (1.10-3.00, *p* = 0.020)
Procedure start time				
0800–1800 (daytime)	1975 (47.5)	162 (44.1)		
1800–2200 (evening)	1057 (25.4)	105 (28.6)	1.21 (0.93–1.56, *p* = 0.144)	
2200–0800 (night-time)	1129 (27.1)	100 (27.2)	1.08 (0.83–1.40, *p* = 0.562)	
Surgical safety checklist used				
No, not available in this hospital	826 (19.9)	108 (29.4)		
No, but available in this hospital	253 (6.1)	45 (12.3)	1.36 (0.93–1.97, *p* = 0.108)	0.92 (0.51–1.65, *p* = 0.771)
Yes	3082 (74.1)	214 (58.3)	0.53 (0.42–0.68, *p* < 0.001)	1.00 (0.64–1.54, *p* = 0.987)
Prophylactic antibiotics				
No	445 (10.7)	43 (11.7)		
Yes	3716 (89.3)	324 (88.3)	0.90 (0.65–1.27, *p* = 0.545)	0.99 (0.66–1.50, *p* = 0.976)
Senior surgeon > 5 years training				
No	943 (22.7)	138 (37.6)		
Yes	3217 (77.3)	229 (62.4)	0.49 (0.39–0.61, *p* < 0.001)	0.86 (0.60–1.23, *p* = 0.395)
Senior anesthetist > 5 years training				
No	1006 (24.2)	154 (42.0)		
Yes	3155 (75.8)	213 (58.0)	0.44 (0.35–0.55, *p* < 0.001)	0.86 (0.60–1.24, *p* = 0.428)
Laparoscopic approach				
No	2338 (56.2)	326 (88.8)		
Yes	1823 (43.8)	41 (11.2)	0.16 (0.11–0.22, *p* < 0.001)	0.22 (0.14–0.33, *p* < 0.001)
Perforated viscus				
No	3654 (87.9)	258 (70.5)		
Yes	502 (12.1)	108 (29.5)	3.05 (2.38–3.88, *p* < 0.001)	3.36 (2.47–4.59, *p* < 0.001)

Data are *n* (%) unless otherwise stated. Hospitals = 339, countries = 52. AIC = 2164. c-statistic = 0.849

*OR* odds ratio, *CI* confidence interval, *HDI* human development index, *ASA* American Association of Anesthesiologists risk score, *SD* standard deviation

There was no association in multilevel models between HDI and OSI (Table S1, Supplemental Digital Content).

Determining any influence of laparoscopy compared with an open approach is difficult given the inherent selection bias and the lower availability in low-HDI and middle-HDI countries. This is highlighted by the different populations undergoing laparoscopic compared with open procedures (Table S2, Supplemental Digital Content). Differences in the laparoscopic group included more females, lower ASA score, a greater likelihood of senior surgeon and anesthetist involvement, and lower perforation rates. Two approaches were taken to attempt to reduce the effect of this imbalance: multilevel logistic regression modeling and a propensity-score matched analysis.

After accounting for case-mix imbalance, laparoscopy was still associated with significantly fewer complications (OR 0.55, 95% CI 0.42–0.71, *p* < 0.001, Table 3) and SSIs (OR 0.22, 95% CI 0.14–0.33, *p* < 0.001, Table 4). To try and communicate these differences more meaningfully, a simple simulation was performed using different patient baseline characteristics (Table 5). This analysis shows an absolute risk reduction (ARR) in overall complication rate associated with laparoscopy of around 6% [number-needed-to-treat (NNT) = 16] in the absence of perforation and 17% (NNT = 8) when the appendix is perforated across HDI groups. For SSI, the analysis implies a greater potential benefit of laparoscopy in low-HDI (ARR 18.6%, 95% CI 11.4–27.7 with perforation) and middle-income countries (ARR 12.2%, 95% CI 6.6–19.6% with perforation), which is expected given the association of HDI and SSI seen in the multilevel model.

**Table 5:** Overall complications (top) and SSI (bottom) simulation

	1	2	3	4	5	6
HDI group Perforation	Low No	Low Yes	Middle No	Middle Yes	High No	High Yes
Overall complications						
Complication (%) with open approach	14.7 (9.7–20.9)	37.0 (26.8–48.3)	13.0 (9.1–17.7)	33.7 (24.8–43.9)	13.6 (9.6–18.3)	35.0 (26.3–44.5)
Complication (%) with lap approach	8.5 (5.1–12.8)	24.0 (15.5–34.5)	7.4 (4.7–10.9)	21.5 (14.1–30.9)	7.8 (5.3–10.7)	22.4 (15.7–30.1)
Absolute risk reduction (%) open versus laparoscopic	6.3 (3.5–9.6)	13.0 (7.7–18.5)	5.6 (3.3–8.2)	12.3 (7.5–17.1)	5.9 (3.3–8.8)	12.6 (7.5–18.1)
Number-needed-to-treat	16 (11–29)	8 (6–13)	18 (13–31)	9 (6–14)	17(12–31)	8 (6–14)
Surgical site infection (SSI)						
SSI (%) with open approach	9.8 (5.5–15.9)	26.1 (15.8–39.2)	5.7 (2.9–9.7)	16.6 (8.9–26.8)	5.1 (2.8–8.0)	15.0 (8.7–23.3)
SSI (%) with lap approach	2.4 (1.1–4.5)	7.5 (3.5–13.6)	1.4 (0.6–2.6)	4.4 (1.9–8.5)	1.2 (0.6–2.1)	3.9 (1.9–6.9)
Absolute risk reduction (%) open versus laparoscopic	7.4 (4.1–12.1)	18.6 (11.4–27.7)	4.8 (2.2–7.4)	12.2 (6.6–19.6)	3.9 (2.1–6.4)	11.2 (6.3–17.4)
Number-needed-to-treat	14 (9–25)	6 (4–9)	21 (14–46)	9 (6–16)	26 (16–48)	9 (6–16)

The multilevel logistic regression models were simulated to convert model coefficients into real-world quantities of interest. The probabilities for different characteristics are shown, together with ARR for open versus laparoscopic. NNT with laparoscopic approach to save a complication. Data are proportion as a percentage (95% CI). Baseline covariate levels are age 29 years, gender male, ASA score 1, diabetes no, currently smoking no, checklist no, antibiotics yes, senior surgeon training > 5 years yes, anesthetics training > 5 years yes

**Fig. 1:**
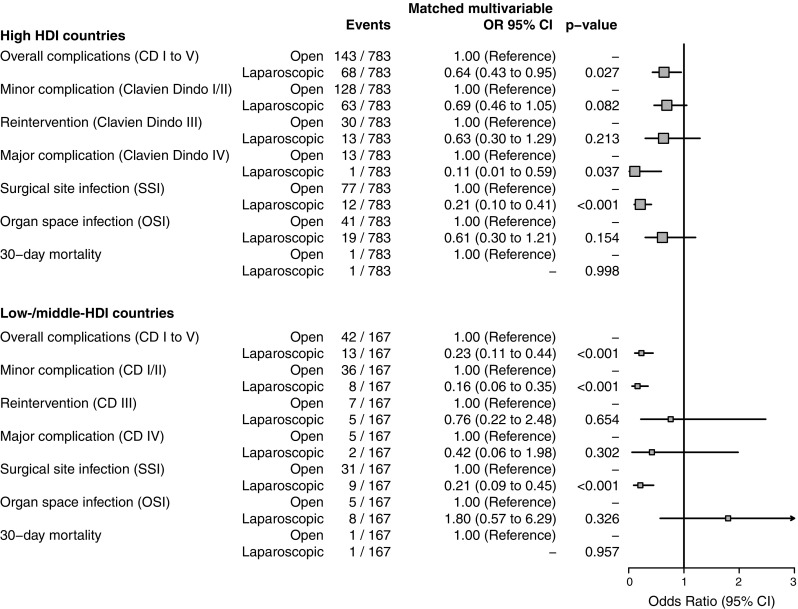
Odds ratio plot for all outcome measures after propensity score matching by HDI group. *OR* odds ratio, *CD* Clavien–Dindo grade, *CI* confidence interval. Note there was an insufficient number of events to specify models for 30-day mortality

To further explore these relationships, a propensity-score-based matching analysis was performed. Patients were first subset into low-/middle-HDI and high-HDI groups. Using all available independent baseline variables and a nearest neighbor approach, 167 patients in the laparoscopic group were matched to 167 in the open group in the low-/middle-HDI subset (Fig. 1, Tables S3 and S4, Supplemental Digital Content) and 783 matched in the high-HDI group. Balance was more easily achieved in the low-/middle-HDI group, with persistent imbalance only seen for seniority of anesthetist and surgical safety checklist use. Balance using nearest neighbor matching was difficult to achieve in the high-HDI group, reflecting de facto differences in the characteristics of patients offered laparoscopic compared with those undergoing open procedures in practice. Alternative matching procedures were successfully explored, but have not been included for space and clarity. Adjusted logistic regression models were applied to the matched sets to address any residual confounding. In the low-/middle-HDI group, a laparoscopic approach was still associated with fewer overall complications (OR 0.23 95% CI 0.11–0.44, *p* < 0.001), minor complications (OR 0.16 95% CI 0.06–0.35, *p* < 0.001), and episodes of SSI (OR 0.21 95% CI 0.09–0.45, *p* < 0.001) (Table S5, Supplemental Digital Content, Fig. 1). Similar results were observed within the matched high-HDI group (Table S6, Supplemental Digital Content).

## Discussion

This is the first patient-level prospective study to describe the surgical approach to appendectomy and the frequency of postoperative complications on a global scale. Our findings reveal clear differences in the management of appendicitis and disparities in complication frequency between different HDI groups, with significantly more wound infections (SSI) in low-HDI versus high-HDI countries. A laparoscopic approach was common in the high-HDI group (1693/2499, 67.7%) but infrequent in low- (41/507, 8.1%) and middle-HDI (132/1540, 8.6%) groups. In analyses that attempted as far as it is possible to account for the inherent selection bias in these observational data, laparoscopy in low-/middle-HDI countries was significantly associated with fewer complications and lower SSI rates.

We have previously shown differences in outcomes after emergency surgery by HDI, with mortality found to be higher in low- and middle-HDI countries compared with high-HDI countries both in adults [4] and children [17]. In the present study, unadjusted analyses show a similar picture. However, in the multivariable analysis for overall complications, no persistent association was seen with HDI after accounting for case-mix and structural differences. There were persistent differences seen in SSI rates after adjustment. SSI is therefore a phenomenon in which factors not accounted for in our models play an important role. More studies are needed to understand how this can be intervened upon.

It is well established in high-income settings that laparoscopic appendectomy is associated with better outcomes; however, little information exists in low-resource settings [18–21]. Wei et al. undertook a randomized comparison of open and laparoscopic appendectomy and concluded that a laparoscopic approach was associated with lower SSI rates and fewer complications,[18] a finding similar to that observed in the current study. Further advantages include reduced postoperative stay, less postoperative pain, and lower oxidative stress [19, 20]. In our study, the presence of a perforated appendix was associated with a 3- to 4-fold increase in adverse outcomes in multivariable models, including an increased surgical site and organ space (abscess) infection, findings similar to previous studies [21]. It has been debated whether a laparoscopic approach is always appropriate for patients with more advanced disease and peritonitis; however, evidence does exist supporting a laparoscopy even in complicated cases where reduced infection rates and postoperative length of stay can be achieved [22, 23].

Appendicitis is common in low-resource settings but its incidence varies greatly by country. It is the fourth-most common procedure performed by training surgeons in the College of Surgeons of East, Central and Southern Africa (COSECSA) region [24]. Appendicitis also presents differently in the poorest countries,[25] which is supported in the current study by the higher perforation rate seen in the low-HDI group (21.5%) compared with high-HDI group (13.9%). Given traditional assumptions around treatment delay and progression to perforation, this may be due to the well-described first-delay in seeking medical attention in low-HDI settings, compounded by a second-delay in reaching a medical facility that may be many days’ travel away [3]. Alternatively, this may represent a pathophysiological distinction between perforated and non-perforated appendicitis,[26] with selection exaggerated in low-resource settings by delays in seeking medical attention arising from geographic barriers to access, cultural differences, and the potential out-of-pocket impoverishing or catastrophic costs of surgery discouraging all but the sickest in attending hospital [27, 28].

The increased use of laparoscopy in low-income settings is controversial. In the poorest regions, the provision of any surgical service is extremely difficult and current high-technology, resource intensive laparoscopy is not feasible. However, our results raise the possibility of a benefit for patients and the healthcare system more widely. Given the higher rates of SSI in some low-income settings, the absolute benefit of laparoscopy is likely to be greater as shown by the simulation results [ARR of SSI in low-HDI countries (18.6%) compared with high-HDI countries (11.2%)]. This translates to a NNT with laparoscopic rather than open surgery of 6 to prevent one SSI in low-HDI countries managing perforated appendicitis. Moreover, in the absence of CT imaging, diagnostic laparoscopy can provide an effective way to investigate patients with acute abdominal pain short of the requirement for a full laparotomy.

The major strength of this study is the collection of patient-level prospective data from 52 countries. This unique dataset allows comparison across the HDI spectrum which is rarely possible. A published, detailed protocol translated into major languages ensured consistent data collection across varying regions without the burden of communication barriers. Local validation by leads ensured data accuracy and completeness was high. The interpretation of observational data is always difficult due to the inherent bias implicit in treatment allocation of patients. We have explored both multilevel models accounting for clustering patients in hospitals and countries, and propensity-score-matched models. In the latter, patients within low-/middle-HDI countries undergoing open or laparoscopic approaches were matched using available baseline variables, thus attempting to compare only “similar” patients who had undergone alternative treatments. Good balance was achieved and benefits of laparoscopy continue to be seen.

There are a number of weaknesses associated with our approach. Denominator data at a country level cannot easily be collected using this methodology. The “snapshot” may not accurately reflect overall practice, particularly where there are prominent seasonal effects [29]. We were not able to capture the degree of sepsis beyond the ASA and perforation rates, which may result in residual confounding. Within low-income, rural settings, collection of 30-day follow-up data can be challenging as patients are often discharged to remote regions and therefore cannot return to attend follow-up clinics. This was minimized by liberal use of telephone follow-up where feasible. A subsequent validation study of this methodology suggests that telephone follow-up is possible in upwards of one-third of included patients. Validation of data entry is challenging particularly where patient record keeping is limited. Collaborators frequently commented that the collected study data were of higher quality than existing hospital data. At each participating center, a lead was appointed to perform data validation and ensure accurate data-entry.

These results have significant implications for policy makers. Variation exists in outcomes after appendectomy across the world, but at least some of that variation may be explained by local surgical infrastructure such as the availability of laparoscopic surgery. Health services are faced with competing priorities and must balance a desire to improve surgical care with the costs of treating, say, malaria. The introduction of any new technology in a resource-poor setting must also be done with care. Recommendations include ensuring the existence of sufficient financial support and organizational systems to address staff training, including biomedical engineering staff to undertake equipment maintenance, and to establish robust, affordable, and sustainable supply chains [10]. A successful implementation of laparoscopic surgery has already been demonstrated at scale in low-resource settings [30].

Future research must focus on identifying innovative, affordable, and safe strategies to implement and scale laparoscopic surgery in low-resource settings. Such adaptive techniques may include gasless laparoscopy, room air insufflation, and development of affordable, reusable instrumentation. In addition, there is an ongoing need to establish robust systems for the continuous measurement of surgical outcomes across the world. High-quality clinical trials relevant to low-HDI countries must be performed to ensure the identification of the most effective treatments, such as how to reduce SSI. Outcomes in surgery will only improve with system-wide improvements across the pre-hospital, hospital, and post-hospital sectors.

This study has shown significant variation in the management and outcomes following appendectomy worldwide. The availability of laparoscopy differs by country HDI, and appears to be significantly associated with better outcomes. There are profound clinical, operational, and financial barriers to the introduction of laparoscopy that if overcome could result in significantly improved outcomes for patients and the wider health system in low-resource environments.

### Electronic supplementary material

Below is the link to the electronic supplementary material.

Supplementary material 1 (DOCX 40 KB)
