# Supplementary material for: Laparoscopy in management of appendicitis in high-, middle-, and low-income countries: a multicenter, prospective, cohort study
Source: Surg Endosc. 2018 Apr 5;32(8):3450–66. doi: 10.1007/s00464-018-6064-9 (PMC6061087; doi:10.1007/s00464-018-6064-9)
Supplement: Supplementary file 1 — Supplementary material 1 (DOCX 40 KB) [file 464_2018_6064_MOESM1_ESM.docx]

# **SUPPLEMENTAL DIGITAL CONTENT**

Table S1. Organ-space infection.

|  |  | Organ-space infection | |  |  |
| --- | --- | --- | --- | --- | --- |
|  |  | No | Yes | Univariable logistic regression  OR (95% CI, P value) | Multilevel logistic regression  OR (95% CI, P value) |
| HDI tertile | High | 2397 (54.8) | 101 (66.0) |  |  |
|  | Middle | 1486 (34.0) | 39 (25.5) | 0.62 (0.42-0.90, p=0.013) | 0.67 (0.36-1.26, p=0.216) |
|  | Low | 494 (11.3) | 13 (8.5) | 0.62 (0.33-1.08, p=0.115) | 0.60 (0.25-1.46, p=0.258) |
| Age in completed years | Mean (SD) | 28.8 (16.5) | 32.5 (20.5) | 1.01 (1.00-1.02, p=0.007) | 1.00 (0.99-1.01, p=0.734) |
| Gender | Male | 2336 (53.4) | 78 (51.0) |  |  |
|  | Female | 2041 (46.6) | 75 (49.0) | 1.10 (0.80-1.52, p=0.560) | 1.21 (0.85-1.73, p=0.283) |
| Diabetes history | No | 4259 (97.3) | 142 (92.8) |  |  |
|  | Yes | 118 (2.7) | 11 (7.2) | 2.80 (1.39-5.08, p=0.002) | 1.82 (0.85-3.91, p=0.122) |
| Smoking currently | No | 3582 (81.9) | 121 (79.1) |  |  |
|  | Yes | 792 (18.1) | 32 (20.9) | 1.20 (0.79-1.76, p=0.377) | 1.00 (0.64-1.58, p=0.996) |
| ASA score | 1 | 3064 (72.0) | 87 (57.2) |  |  |
|  | 2 | 994 (23.4) | 52 (34.2) | 1.84 (1.29-2.61, p=0.001) | 1.39 (0.91-2.11, p=0.131) |
|  | >=3 | 198 (4.7) | 13 (8.6) | 2.31 (1.21-4.07, p=0.006) | 1.44 (0.68-3.07, p=0.343) |
| Procedure start time | 0800-1800 (daytime) | 2059 (47.0) | 79 (51.6) |  |  |
|  | 1800-2200 (evening) | 1122 (25.6) | 41 (26.8) | 0.95 (0.64-1.39, p=0.804) |  |
|  | 2200-0800 (night-time) | 1196 (27.3) | 33 (21.6) | 0.72 (0.47-1.08, p=0.117) |  |
| Surgical safety checklist used | No, not available in this hospital | 914 (20.9) | 20 (13.1) |  |  |
|  | No, but available in this hospital | 290 (6.6) | 8 (5.2) | 1.26 (0.52-2.79, p=0.585) | 1.04 (0.38-2.88, p=0.940) |
|  | Yes | 3173 (72.5) | 125 (81.7) | 1.80 (1.14-2.99, p=0.016) | 1.04 (0.54-2.01, p=0.899) |
| Prophylactic antibiotics | No | 472 (10.8) | 16 (10.5) |  |  |
|  | Yes | 3905 (89.2) | 137 (89.5) | 1.03 (0.63-1.82, p=0.898) | 1.01 (0.55-1.85, p=0.976) |
| Senior surgeon >5 years training | No | 1059 (24.2) | 23 (15.0) |  |  |
|  | Yes | 3317 (75.8) | 130 (85.0) | 1.80 (1.18-2.90, p=0.010) | 0.92 (0.49-1.71, p=0.781) |
| Senior anesthetist >5 years training | No | 1140 (26.0) | 21 (13.7) |  |  |
|  | Yes | 3237 (74.0) | 132 (86.3) | 2.21 (1.42-3.62, p=0.001) | 1.69 (0.89-3.21, p=0.112) |
| Laparoscopic approach | No | 2581 (59.0) | 85 (55.6) |  |  |
|  | Yes | 1796 (41.0) | 68 (44.4) | 1.15 (0.83-1.59, p=0.400) | 0.86 (0.55-1.33, p=0.487) |
| Perforated viscus | No | 3828 (87.6) | 85 (55.6) |  |  |
|  | Yes | 543 (12.4) | 68 (44.4) | 5.64 (4.04-7.85, p<0.001) | 5.51 (3.74-8.12, p<0.001) |

Complete data. Data are n (%) unless otherwise stated. Hospitals=339, countries=52. AIC=1229. c-statistic=0.883. OR odds ratio, CI confidence interval, HDI human development index, ASA American Association of Anesthesiologists risk score, SD standard deviation.

Table S2. Patient and operative characteristics by operative approach.

|  |  | Laparoscopic approach | |  |
| --- | --- | --- | --- | --- |
|  |  | No | Yes | p-value |
| HDI tertile | High | 806 (30.1) | 1693 (90.7) | <0.001 |
|  | Middle | 1408 (52.5) | 132 (7.1) |  |
|  | Low | 466 (17.4) | 41 (2.2) |  |
| Age in completed years | Mean (SD) | 27.8 (16.4) | 30.7 (16.8) | <0.001 |
| Gender | Male | 1521 (56.8) | 901 (48.3) | <0.001 |
|  | Female | 1159 (43.2) | 965 (51.7) |  |
| Diabetes history | No | 2598 (96.9) | 1819 (97.5) | 0.280 |
|  | Yes | 82 (3.1) | 47 (2.5) |  |
| Smoking currently | No | 2220 (82.8) | 1498 (80.3) | 0.027 |
|  | Yes | 458 (17.1) | 367 (19.7) |  |
|  | Missing | 2 (0.1) | 1 (0.1) |  |
| ASA score | 1 | 1854 (69.2) | 1312 (70.3) | <0.001 |
|  | 2 | 592 (22.1) | 455 (24.4) |  |
|  | >=3 | 154 (5.7) | 57 (3.1) |  |
|  | Missing | 80 (3.0) | 42 (2.3) |  |
| Procedure start time | 0800-1800 (daytime) | 1095 (40.9) | 1053 (56.4) | <0.001 |
|  | 1800-2200 (evening) | 726 (27.1) | 439 (23.5) |  |
|  | 2200-0800 (night-time) | 859 (32.1) | 374 (20.0) |  |
| Surgical safety checklist used | No, not available in this hospital | 856 (31.9) | 83 (4.4) | <0.001 |
|  | No, but available in this hospital | 275 (10.3) | 24 (1.3) |  |
|  | Yes | 1549 (57.8) | 1759 (94.3) |  |
| Prophylactic antibiotics | No | 316 (11.8) | 174 (9.3) | 0.008 |
|  | Yes | 2364 (88.2) | 1692 (90.7) |  |
|  | Missing | 0 (0.0) | 1 (0.1) |  |
| Senior surgeon >5 years training | No | 988 (36.9) | 99 (5.3) | <0.001 |
|  | Yes | 1691 (63.1) | 1767 (94.7) |  |
|  | Missing | 1 (0.0) | 0 (0.0) |  |
| Senior anesthetist >5 years training | No | 1062 (39.6) | 107 (5.7) | <0.001 |
|  | Yes | 1618 (60.4) | 1759 (94.3) |  |
| Perforated viscus | No | 2289 (85.4) | 1635 (87.6) | 0.051 |
|  | Yes | 385 (14.4) | 231 (12.4) |  |
|  | Missing | 6 (0.2) | 0 (0.0) |  |

Data are n (%) unless otherwise stated. SD, standard deviation. OR odds ratio, CI confidence interval, HDI human development index, ASA American Association of Anesthesiologists risk score.

Table S3. Pre-propensity score matching.

|  |  | Laparoscopic approach (pre-propensity score match) | | | | | | |
| --- | --- | --- | --- | --- | --- | --- | --- | --- |
|  |  | High HDI |  |  |  | Low-Middle HDI | |  |
|  |  | Open | Laparoscopic | p-value |  | Open | Laparoscopic | p-value |
| HDI tertile | High | 806 (100.0) | 1693 (100.0) | - |  | 0 (0.0) | 0 (0.0) | 0.734 |
|  | Middle | - | - |  |  | 1408 (75.1) | 132 (76.3) |  |
|  | Low | - | - |  |  | 466 (24.9) | 41 (23.7) |  |
| Age in completed years | Mean (SD) | 32.4 (20.9) | 30.6 (16.7) | 0.885 |  | 25.7 (13.6) | 31 (17.6) | <0.001 |
| Gender | Male | 523 (64.9) | 821 (48.5) | <0.001 |  | 998 (53.3) | 80 (46.2) | 0.077 |
|  | Female | 283 (35.1) | 872 (51.5) |  |  | 876 (46.7) | 93 (53.8) |  |
| Diabetes history | No | 781 (96.9) | 1654 (97.7) | 0.238 |  | 1817 (97.0) | 165 (95.4) | 0.256 |
|  | Yes | 25 (3.1) | 39 (2.3) |  |  | 57 (3.0) | 8 (4.6) |  |
| Smoking currently | No | 637 (79.0) | 1340 (79.2) | 0.925 |  | 1583 (84.6) | 158 (91.3) | 0.017 |
|  | Yes | 169 (21.0) | 352 (20.8) |  |  | 289 (15.4) | 15 (8.7) |  |
| ASA score | 1 | 517 (65.9) | 1207 (72.9) | <0.001 |  | 1337 (73.7) | 105 (62.5) | 0.005 |
|  | 2 | 211 (26.9) | 402 (24.3) |  |  | 381 (21.0) | 53 (31.5) |  |
|  | >=3 | 57 (7.3) | 47 (2.8) |  |  | 97 (5.3) | 10 (6.0) |  |
| Admission to procedure time | < 6 hours | 218 (27.1) | 334 (19.7) | <0.001 |  | 957 (51.1) | 43 (24.9) | <0.001 |
|  | 6-11 hours | 207 (25.7) | 437 (25.8) |  |  | 530 (28.3) | 57 (32.9) |  |
|  | 12-23 hour | 241 (29.9) | 531 (31.4) |  |  | 289 (15.4) | 48 (27.7) |  |
|  | 24-47 hours | 107 (13.3) | 274 (16.2) |  |  | 71 (3.8) | 20 (11.6) |  |
|  | 48+ hours | 32 (4.0) | 116 (6.9) |  |  | 26 (1.4) | 5 (2.9) |  |
| Procedure start time | 0800-1800 (daytime) | 380 (47.1) | 965 (57.0) | <0.001 |  | 715 (38.2) | 88 (50.9) | 0.004 |
|  | 1800-2200 (evening) | 218 (27.0) | 398 (23.5) |  |  | 508 (27.1) | 41 (23.7) |  |
|  | 2200-0800 (night-time) | 208 (25.8) | 330 (19.5) |  |  | 651 (34.7) | 44 (25.4) |  |
| Surgical safety checklist used | No, not available in this hospital | 112 (13.9) | 70 (4.1) | <0.001 |  | 744 (39.7) | 13 (7.5) | <0.001 |
|  | No, but available in this hospital | 18 (2.2) | 17 (1.0) |  |  | 257 (13.7) | 7 (4.0) |  |
|  | Yes | 676 (83.9) | 1606 (94.9) |  |  | 873 (46.6) | 153 (88.4) |  |
| Prophylactic antibiotics | No | 62 (7.7) | 161 (9.5) | 0.136 |  | 254 (13.6) | 13 (7.5) | 0.024 |
|  | Yes | 744 (92.3) | 1532 (90.5) |  |  | 1620 (86.4) | 160 (92.5) |  |
| Senior surgeon >5 years training | No | 43 (5.3) | 65 (3.8) | 0.086 |  | 945 (50.5) | 34 (19.7) | <0.001 |
|  | Yes | 763 (94.7) | 1628 (96.2) |  |  | 928 (49.5) | 139 (80.3) |  |
| Senior anaesthetist >5 years training | No | 38 (4.7) | 77 (4.5) | 0.853 |  | 1024 (54.6) | 30 (17.3) | <0.001 |
|  | Yes | 768 (95.3) | 1616 (95.5) |  |  | 850 (45.4) | 143 (82.7) |  |
| Perforated viscus | No | 653 (81.1) | 1497 (88.4) | <0.001 |  | 1636 (87.5) | 138 (79.8) | 0.004 |
|  | Yes | 152 (18.9) | 196 (11.6) |  |  | 233 (12.5) | 35 (20.2) |  |
| Overall Complications (Clavien Dindo I, II, III, IV,V) | No | 663 (82.3) | 1519 (89.7) | <0.001 |  | 1559 (83.2) | 158 (91.9) | 0.003 |
|  | Yes | 143 (17.7) | 174 (10.3) | <0.001 |  | 314 (16.8) | 14 (8.1) |  |
| Minor complication (Clavien Dindo I/II) | No | 678 (84.1) | 1541 (91.1) | <0.001 |  | 1574 (84.8) | 163 (94.8) | <0.001 |
|  | Yes | 128 (15.9) | 150 (8.9) | <0.001 |  | 283 (15.2) | 9 (5.2) |  |
| Reintervention  (Clavien Dindo III) | No | 776 (96.3) | 1655 (97.8) | 0.034 |  | 1828 (97.6) | 167 (97.1) | 0.682 |
|  | Yes | 30 (3.7) | 38 (2.2) | 0.034 |  | 45 (2.4) | 5 (2.9) |  |
| Major complication (Clavien Dindo IV) | No | 793 (98.4) | 1681 (99.3) | 0.034 |  | 1848 (98.7) | 170 (98.8) | 0.850 |
|  | Yes | 13 (1.6) | 12 (0.7) | 0.034 |  | 25 (1.3) | 2 (1.2) |  |
| 30-day mortality (presumed) | Alive | 805 (99.9) | 1691 (99.9) | 0.968 |  | 1865 (99.8) | 171 (99.4) | 0.351 |
|  | Died | 1 (0.1) | 2 (0.1) | 0.968 |  | 4 (0.2) | 1 (0.6) |  |
| Surgical site infection (SSI) | No | 729 (90.4) | 1660 (98.1) | <0.001 |  | 1609 (86.6) | 163 (94.8) | 0.002 |
|  | Yes | 77 (9.6) | 32 (1.9) | <0.001 |  | 249 (13.4) | 9 (5.2) |  |
| Organ space infection (OSI) | No | 765 (94.9) | 1632 (96.5) | 0.068 |  | 1816 (97.6) | 164 (95.3) | 0.069 |
|  | Yes | 41 (5.1) | 60 (3.5) | 0.068 |  | 44 (2.4) | 8 (4.7) |  |

OR odds ratio, CI confidence interval, HDI human development index, ASA American Association of Anesthesiologists risk score, SD standard deviation.

Table S4. Post-propensity score matching

|  |  | Laparoscopic approach (post-propensity score match) | | | | | | |
| --- | --- | --- | --- | --- | --- | --- | --- | --- |
|  |  | High HDI |  |  |  | Low-Middle HDI | | |
|  |  | Open | Laparoscopic | p-value |  | Open | Laparoscopic | p-value |
| HDI tertile | High | 783 (100.0) | 783 (100.0) | NA |  | 0 (0.0) | 0 (0.0) | 1.000 |
|  | Middle | 0 (0.0) | 0 (0.0) | NA |  | 126 (75.4) | 126 (75.4) |  |
|  | Low | 0 (0.0) | 0 (0.0) | NA |  | 41 (24.6) | 41 (24.6) |  |
| Age in completed years | Mean (SD) | 32.4 (20.9) | 29.5 (15) | 0.502 |  | 28.9 (16.4) | 30.9 (17.1) | 0.201 |
| Gender | Male | 505 (64.5) | 62 (7.9) | <0.001 |  | 80 (47.9) | 77 (46.1) | 0.742 |
|  | Female | 278 (35.5) | 721 (92.1) | <0.001 |  | 87 (52.1) | 90 (53.9) |  |
| Diabetes history | No | 758 (96.8) | 772 (98.6) | 0.018 |  | 161 (96.4) | 160 (95.8) | 0.777 |
|  | Yes | 25 (3.2) | 11 (1.4) | 0.018 |  | 6 (3.6) | 7 (4.2) |  |
| Smoking currently | No | 622 (79.4) | 601 (76.8) | 0.199 |  | 158 (94.6) | 152 (91.0) | 0.204 |
|  | Yes | 161 (20.6) | 182 (23.2) | 0.199 |  | 9 (5.4) | 15 (9.0) |  |
| ASA score | 1 | 516 (65.9) | 606 (77.4) | <0.001 |  | 105 (62.9) | 105 (62.9) | 0.563 |
|  | 2 | 210 (26.8) | 177 (22.6) | <0.001 |  | 56 (33.5) | 52 (31.1) |  |
|  | >=3 | 57 (7.3) | 0 (0.0) | <0.001 |  | 6 (3.6) | 10 (6.0) |  |
| Admission to procedure time | < 6 hours | 213 (27.2) | 101 (12.9) | <0.001 |  | 42 (25.1) | 40 (24.0) | 0.852 |
|  | 6-11 hours | 202 (25.8) | 177 (22.6) | <0.001 |  | 49 (29.3) | 57 (34.1) |  |
|  | 12-23 hour | 233 (29.8) | 249 (31.8) | <0.001 |  | 52 (31.1) | 45 (26.9) |  |
|  | 24-47 hours | 104 (13.3) | 151 (19.3) | <0.001 |  | 18 (10.8) | 20 (12.0) |  |
|  | 48+ hours | 31 (4.0) | 105 (13.4) | <0.001 |  | 6 (3.6) | 5 (3.0) |  |
| Procedure start time | 0800-1800 (daytime) | 369 (47.1) | 459 (58.6) | <0.001 |  | 64 (38.3) | 85 (50.9) | 0.069 |
|  | 1800-2200 (evening) | 210 (26.8) | 177 (22.6) | <0.001 |  | 48 (28.7) | 39 (23.4) |  |
|  | 2200-0800 (night-time) | 204 (26.1) | 147 (18.8) | <0.001 |  | 55 (32.9) | 43 (25.7) |  |
| Surgical safety checklist used | No, not available in this hospital | 108 (13.8) | 25 (3.2) | <0.001 |  | 61 (36.5) | 13 (7.8) | <0.001 |
|  | No, but available in this hospital | 18 (2.3) | 7 (0.9) | <0.001 |  | 22 (13.2) | 6 (3.6) |  |
|  | Yes | 657 (83.9) | 751 (95.9) | <0.001 |  | 84 (50.3) | 148 (88.6) |  |
| Prophylactic antibiotics | No | 60 (7.7) | 87 (11.1) | 0.019 |  | 24 (14.4) | 12 (7.2) | 0.034 |
|  | Yes | 723 (92.3) | 696 (88.9) | 0.019 |  | 143 (85.6) | 155 (92.8) |  |
| Senior surgeon >5 years training | No | 43 (5.5) | 23 (2.9) | 0.012 |  | 69 (41.3) | 34 (20.4) | <0.001 |
|  | Yes | 740 (94.5) | 760 (97.1) | 0.012 |  | 98 (58.7) | 133 (79.6) |  |
| Senior anaesthetist >5 years training | No | 37 (4.7) | 36 (4.6) | 0.905 |  | 87 (52.1) | 30 (18.0) | <0.001 |
|  | Yes | 746 (95.3) | 747 (95.4) | 0.905 |  | 80 (47.9) | 137 (82.0) |  |
| Perforated viscus | No | 633 (80.8) | 764 (97.6) | <0.001 |  | 134 (80.2) | 133 (79.6) | 0.891 |
|  | Yes | 150 (19.2) | 19 (2.4) | <0.001 |  | 33 (19.8) | 34 (20.4) |  |
|  |  |  |  |  |  |  |  |  |
| Overall Complications | No | 640 (81.7) | 715 (91.3) | <0.001 |  | 125 (74.9) | 154 (92.2) | <0.001 |
|  | Yes | 143 (18.3) | 68 (8.7) | <0.001 |  | 42 (25.1) | 13 (7.8) |  |
| Minor complication (Clavien Dindo I/II) | No | 655 (83.7) | 720 (92.0) | <0.001 |  | 131 (78.4) | 159 (95.2) | <0.001 |
|  | Yes | 128 (16.3) | 63 (8.0) | <0.001 |  | 36 (21.6) | 8 (4.8) |  |
| Reintervention (Clavien Dindo III) | No | 753 (96.2) | 770 (98.3) | 0.009 |  | 160 (95.8) | 162 (97.0) | 0.557 |
|  | Yes | 30 (3.8) | 13 (1.7) | 0.009 |  | 7 (4.2) | 5 (3.0) |  |
| Major complication/Reintervention (Clavien Dindo III/IV) | No | 770 (98.3) | 782 (99.9) | 0.001 |  | 162 (97.0) | 165 (98.8) | 0.252 |
|  | Yes | 13 (1.7) | 1 (0.1) | 0.001 |  | 5 (3.0) | 2 (1.2) |  |
| 30-day mortality (presumed) | Alive | 782 (99.9) | 782 (99.9) | 1.000 |  | 166 (99.4) | 166 (99.4) | 1.000 |
|  | Died | 1 (0.1) | 1 (0.1) | 1.000 |  | 1 (0.6) | 1 (0.6) |  |
| Surgical site infection (SSI) | No | 706 (90.2) | 771 (98.5) | <0.001 |  | 136 (81.4) | 158 (94.6) | <0.001 |
|  | Yes | 77 (9.8) | 12 (1.5) | <0.001 |  | 31 (18.6) | 9 (5.4) |  |
| Organ space infection (OSI) | No | 742 (94.8) | 764 (97.6) | 0.004 |  | 162 (97.0) | 159 (95.2) | 0.396 |
|  | Yes | 41 (5.2) | 19 (2.4) | 0.004 |  | 5 (3.0) | 8 (4.8) |  |

OR odds ratio, CI confidence interval, HDI human development index, ASA American Association of Anesthesiologists risk score, SD standard deviation.

Table S5. Propensity-matched analysis for low- and middle-HDI countries.

|  |  | Overall complications  (Clavien-Dindo I-V | | Minor complication (Clavien Dindo I-II) | | Reintervention  (Clavien Dindo III) | | Major complication (Clavien Dindo IV) | | Surgical site infection (SSI) | | Organ space infection | |
| --- | --- | --- | --- | --- | --- | --- | --- | --- | --- | --- | --- | --- | --- |
|  |  | OR 95% CI | p-value | OR 95% CI | p-value | OR 95% CI | p-value | OR 95% CI | p-value | OR 95% CI | p-value | OR 95% CI | p-value |
| Approach | Open | - | - | - | - | - | - | - | - | - | - | - | - |
|  | Laparoscopic | 0.23 (0.11 to 0.44) | <0.001 | 0.16 (0.06 to 0.35) | <0.001 | 0.76 (0.22 to 2.48) | 0.654 | 0.42 (0.06 to 1.98) | 0.302 | 0.21 (0.09 to 0.45) | <0.001 | 1.80 (0.57 to 6.29) | 0.326 |
| Age | Per year increase (centred) | 1.00 (0.98 to 1.02) | 0.958 | 1.00 (0.98 to 1.02) | 0.807 | 0.95 (0.90 to 1.00) | 0.059 | 0.97 (0.90 to 1.02) | 0.272 | 1.02 (0.99 to 1.04) | 0.134 | 0.97 (0.93 to 1.01) | 0.157 |
| Gender | Male | - | - | - | - | - | - | - | - | - | - | - | - |
|  | Female | 1.52 (0.80 to 2.97) | 0.209 | 1.19 (0.58 to 2.48) | 0.637 | - | - | - | - | 1.41 (0.68 to 3.02) | 0.362 | 1.63 (0.51 to 5.71) | 0.415 |
| Smoking status | Non-smoker | - | - | - | - | - | - | - | - | - | - | - | - |
|  | Current Smoker | 1.40 (0.35 to 4.59) | 0.603 | 1.72 (0.41 to 6.07) | 0.418 | - | - | - | - | 1.86 (0.45 to 6.50) | 0.355 | - | - |
| ASA | I | - | - | - | - | - | - | - | - | - | - | - | - |
|  | II | 1.10 (0.55 to 2.18) | 0.778 | 0.96 (0.43 to 2.05) | 0.908 | 1.53 (0.38 to 5.38) | 0.515 | - | - | 0.46 (0.19 to 1.05) | 0.075 | 1.31 (0.32 to 4.72) | 0.691 |
|  | >= III | 1.83 (0.40 to 7.14) | 0.402 | 2.23 (0.46 to 9.59) | 0.291 | 4.65 (0.22 to 37.09) | 0.197 | - | - | 0.99 (0.18 to 4.17) | 0.985 | 6.64 (0.80 to 39.88) | 0.048 |
| Perforated | No | - | - | - | - | - | - | - | - | - | - | - | - |
|  | Yes | 3.07 (1.54 to 6.09) | 0.001 | 3.70 (1.76 to 7.77) | 0.001 | 1.44 (0.31 to 5.10) | 0.600 | 1.78 (0.25 to 8.59) | 0.499 | 2.69 (1.22 to 5.79) | 0.012 | 3.02 (0.86 to 9.84) | 0.069 |

Table S6. Propensity-matched analysis for high-HDI countries.

|  |  | Overall complications  (Clavien-Dindo I-V) | | Minor complication (Clavien Dindo I-II) | | Reintervention  (Clavien Dindo III) | | Major complication (Clavien Dindo IV) | | Surgical site infection (SSI) | | Organ space infection | |
| --- | --- | --- | --- | --- | --- | --- | --- | --- | --- | --- | --- | --- | --- |
|  |  | OR 95% CI | p-value | OR 95% CI | p-value | OR 95% CI | p-value | OR 95% CI | p-value | OR 95% CI | p-value | OR 95% CI | p-value |
| Approach | Open | - | - | - | - | - | - | - | - | - | - | - | - |
|  | Laparoscopic | 0.64 (0.43 to 0.95) | 0.027 | 0.69 (0.46 to 1.05) | 0.082 | 0.63 (0.30 to 1.29) | 0.213 | 0.11 (0.01 to 0.59) | 0.037 | 0.21 (0.10 to 0.41) | <0.001 | 0.61 (0.30 to 1.21) | 0.154 |
| Age | Per year increase (centred) | 1.01 (1.00 to 1.02) | 0.023 | 1.01 (1.00 to 1.02) | 0.007 | 1.00 (0.98 to 1.02) | 0.752 | 1.04 (1.02 to 1.07) | <0.001 | 1.01 (1.00 to 1.02) | 0.096 | 1.01 (0.99 to 1.03) | 0.189 |
| Gender | Male | - | - | - | - | - | - | - | - | - | - | - | - |
|  | Female | 1.04 (0.72 to 1.51) | 0.836 | 0.99 (0.68 to 1.46) | 0.978 | - | - | - | - | 1.03 (0.63 to 1.69) | 0.894 | 1.20 (0.64 to 2.23) | 0.568 |
| Smoking status | Non-smoker | - | - | - | - | - | - | - | - | - | - | - | - |
|  | Current Smoker | 1.17 (0.81 to 1.67) | 0.391 | 1.20 (0.82 to 1.73) | 0.343 | - | - | - | - | 0.97 (0.56 to 1.64) | 0.913 | - | - |
| ASA | I | - | - | - | - | - | - | - | - | - | - | - | - |
|  | II | 1.23 (0.85 to 1.77) | 0.273 | 1.21 (0.82 to 1.77) | 0.323 | 1.15 (0.53 to 2.37) | 0.710 | - | - | 1.27 (0.73 to 2.17) | 0.384 | 1.24 (0.65 to 2.28) | 0.499 |
|  | >= III | 2.04 (1.01 to 4.09) | 0.046 | 1.67 (0.80 to 3.40) | 0.165 | 1.47 (0.35 to 5.13) | 0.566 | - | - | 2.17 (0.91 to 5.05) | 0.075 | 0.84 (0.21 to 2.71) | 0.782 |
| Perforated | No | - | - | - | - | - | - | - | - | - | - | - | - |
|  | Yes | 3.87 (2.63 to 5.67) | <0.001 | 3.91 (2.63 to 5.79) | <0.001 | 3.64 (1.75 to 7.38) | <0.001 | 1.25 (0.33 to 3.95) | 0.716 | 2.78 (1.67 to 4.56) | <0.001 | 3.73 (1.99 to 6.86) | <0.001 |

Bayesian simulation method

The multilevel models were simulated to convert model coefficients into real-world quantities of interest. For reasons of computational efficiency, a hierarchical multivariable Bayesian logistic regression model was used. Non-informative priors were used with priors for fixed-effect coefficients normal(0, 10) and random effects coefficients normal(0, sigma), with sigma ~ uniform(0, 100). Sensitivity analyses were performed on alternative priors with no significant change in direction or magnitude of effects. Markov chain Monte Carlo specification was 10000 iterations across 4 chains (40000 total) with 5000 burn-in steps. Different chain initiation points and chain lengths were tested. Convergence was achieved within 10000 iterations and models were not sensitive to different starting points. Model diagnostic plots included traceplots to ensure good mixing of chains, Gelman-Rubin-Brooks plots to show the Gelman-Rubin convergence (Rhat or shrink factor), autocorrelation plots, and posterior density plots for each chain.
